# CAF-derived BHB modulates FXR1-Kbhb and NK-cell lipid metabolism in osteosarcoma

**DOI:** 10.7150/ijbs.128764

**Published:** 2026-09-02

**Authors:** Yihan Yu, Wei Wu, Anzhen Ju, Lei Zhao, Guohui Liang, Yun Liu, Zengwu Shao, Feifei Pu, Binlong Zhong, Fengxia Chen, Jianxiang Liu, Zhicai Zhang

**Affiliations:** 1 Department of Orthopedics, Union Hospital, Tongji Medical College, Huazhong University of Science and Technology, Wuhan 430022, China.; 2 Department of Orthopaedics, Traditional Chinese and Western Medicine Hospital, Hubei University of Chinese Medicine, Wuhan 430022, Hubei, China.; 3 Department of Orthopaedics, Traditional Chinese and Western Medicine Hospital of Wuhan, Tongji Medical College, Huazhong University of Science and Technology, Wuhan 430022, Hubei, China.; 4 Department of Radiation and Medical Oncology, Zhongnan Hospital of Wuhan University, 125 Donghu road, Wuhan, Hubei, China.

**Keywords:** osteosarcoma, natural killer (NK) cells, cancer-associated fibroblasts (CAFs), lipid metabolism, β-hydroxybutyrate (BHB)

## Abstract

Osteosarcoma (OS) is the most common primary malignant bone tumor in children and adolescents and is characterized by high aggressiveness and poor prognosis. Although surgery and chemotherapy have improved overall survival, outcomes for relapsed or metastatic disease remain extremely poor, thus underscoring the urgent need for novel therapeutic strategies. Natural killer (NK) cells are key cytotoxic effectors in tumor immunity, but their function is profoundly suppressed within the OS tumor microenvironment. Furthermore, the role of cancer-associated fibroblasts (CAFs) in mediating NK-cell dysfunction through metabolic regulation remains insufficiently understood. We established coculture systems of NK cells, OS cells, and CAFs and assessed NK-cell activation and cytotoxicity by using functional assays, flow cytometry, and immunofluorescence. Metabolic profiling was performed with extracellular acidification rate, oxygen consumption rate, reactive oxygen species detection, MitoTracker staining, and electron microscopy. Integrated proteomics and mechanistic studies were used to identify CAF-derived metabolites and their effects on NK-cell metabolism. CAFs markedly impaired NK-cell recognition and cytotoxicity toward OS cells, accompanied by metabolic reprogramming characterized by enhanced glycolysis, increased reactive oxygen species production, and reduced mitochondrial activity. Multiomics and functional analyses identified β-hydroxybutyrate (BHB) as a key CAF-derived metabolite that contributed to reduced NK-cell FAO and effector function. BHB treatment increased FXR1 Kbhb and reduced FAO, cytokine secretion, and tumor-cell killing, whereas FXR1 K56 mutation attenuated these functional changes. *In vivo*, CAF-CM treatment was accompanied by elevated circulating BHB, reduced NK-cell activation, and accelerated tumor growth. Targeting the CAF-BHB- FXR1 axis may represent a promising therapeutic strategy to restore NK cell-mediated antitumor immunity and improve treatment outcomes.

## Introduction

Osteosarcoma (OS) is the most common primary malignant bone tumor and predominantly affects children and adolescents 1. It typically originates from mesenchymal stem cells with osteogenic differentiation and preferentially arises in the metaphyseal regions of long bones, such as the distal femur, proximal tibia, and proximal humerus 2,3. The annual incidence of OS is estimated at three to five cases per million. Despite its relatively low frequency, OS carries a high risk of disability and metastasis. At diagnosis, 15%-20% of patients already present with distant metastases (most commonly to the lungs); once metastatic spread occurs either at presentation or relapse, the prognosis is dismal, with five-year survival rates of only 20%-30% 4. Although the current multimodal regimen of neoadjuvant chemotherapy, surgical resection, and adjuvant chemotherapy has significantly improved overall survival, the outcomes for relapsed or metastatic OS remain poor 5. These challenges underscore the urgent need for innovative therapeutic strategies, particularly those harnessing the immune system.

The tumor microenvironment (TME) constitutes a highly heterogeneous ecosystem comprising tumor cells, stromal cells, extracellular matrix (ECM), and soluble factors and is shaped by hypoxia, nutrient deprivation, and the accumulation of metabolic byproducts such as lactate and adenosine 6-8. These features collectively enable tumors to evade immune surveillance, sustain proliferation, and promote metastatic dissemination 9-11. Among the immune cells within the TME, tumor-associated macrophages, natural killer (NK) cells, T cells, and B cells are central players in tumor progression and are key targets for antitumor immunotherapy 12-14.

NK cells, as innate cytotoxic lymphocytes, play a pivotal role in tumor immune surveillance and clearance 15,16. They directly kill malignant cells via the release of perforin, granzymes, tumor necrosis factor alpha, and Fas ligand-mediated apoptotic signaling and indirectly amplify antitumor immunity by secreting cytokines and chemokines that regulate dendritic cells and T cells 17,18. However, NK cells in the OS TME are often functionally suppressed, with impaired activation of receptors such as the NKG2 family, thus resulting in markedly reduced immune surveillance. Therefore, targeting NK-cell checkpoints and metabolic pathways holds promise for restoring their antitumor activity.

In addition to immune cells, cancer-associated fibroblasts (CAFs) represent a major stromal component that actively shapes the OS microenvironment. CAFs promote tumor progression through the secretion of ECM proteins, intercellular signaling, and growth factors and have been shown to drive disease progression in recurrent OS 19. By depositing collagen and other ECM constituents, CAFs create a dense physical barrier that remodels the TME and impedes therapeutic penetration. Under hypoxic conditions, they further release immunosuppressive cytokines such as interleukin 6 and transforming growth factor beta, thereby impairing immune cell cytotoxicity 20.

A hallmark of cancer is metabolic reprogramming, whereby tumors alter their metabolic networks or exploit TME-derived metabolites to sustain growth and biosynthesis 21. CAFs are metabolically active, displaying enhanced autophagy and catabolism that facilitate the release of lactate, ketone bodies, and glutamine, which in turn fuel tumor metabolism and metastasis 22. For example, the loss of caveolin-1 in CAFs augments metabolite release, thus fostering a tumor-favoring metabolic milieu 23. In parallel, CAFs activate signaling pathways such as focal adhesion kinase 1 to enhance glycolysis and redox homeostasis in tumor cells, further boosting invasion. Beyond supporting tumor metabolism, the CAF-mediated remodeling of the metabolic landscape has been shown to directly suppress T- and NK-cell effector functions, thus contributing to immune evasion 24-27. For example, myCAF-derived exosomal PWAR6 enhances tumor cell glutamine uptake, thereby depleting glutamine availability and suppressing NK cell function in the tumor microenvironment 28. Although lactate, adenosine, and glutamine have been widely recognized as classical immunosuppressive metabolites in the TME, the role of CAF-derived ketone bodies in regulating NK-cell function remains much less understood. Among these metabolites, β-hydroxybutyrate (BHB) is particularly relevant because it can act not only as an alternative metabolic substrate but also as an epigenetic regulator through lysine β-hydroxybutyrylation. This dual metabolic and epigenetic property provides a rationale for investigating whether BHB links CAF metabolic remodeling to NK-cell lipid metabolic dysfunction in osteosarcoma.

Despite these advances, it is unclear whether the CAF-mediated suppression of NK cells involves the regulation of lipid metabolism. NK-cell effector functions are highly dependent on precise lipid metabolic control, which is essential for maintaining activation, cytotoxicity, and memory-like phenotypes. Therefore, elucidating how CAF-derived metabolites influence NK-cell lipid metabolism and functional competence has considerable scientific relevance.

By building on this rationale, this study focuses on NK cells with intrinsic antitumor potential within the OS TME. We aim to systematically investigate the role of CAFs and their secreted metabolites in reprogramming NK-cell lipid metabolism and suppressing their effector function. Based on the emerging role of ketone bodies in metabolic-epigenetic regulation, we hypothesized that CAF-derived BHB, rather than serving only as an energy substrate, epigenetically reprograms NK-cell lipid metabolism, leading to impaired fatty acid oxidation and antitumor immunity. By examining the relationship among CAFs, BHB, and NK-cell lipid metabolism, this study evaluates a candidate stromal-metabolic pathway in osteosarcoma.

## Materials and Methods

### Cell culture and treatment

All human-derived cell lines, including two OS-cell lines (143B and KHOS) and a human NK-cell line (NK-92) were obtained from the Cell Bank of the Chinese Academy of Sciences (Shanghai, China). Human CAFs were isolated from fresh osteosarcoma specimens as described below. 143B cells were maintained in McCoy's 5A medium with 10% FBS (Gibco, USA). KHOS cells were cultured in αMEM supplemented with 10% FBS (Gibco, USA). NK-92 cells were cultured in alpha minimum essential medium supplemented with 12.5% FBS (Gibco, USA), 12.5% horse serum (Gibco, USA), 0.2 mM inositol (Sigma-Aldrich, USA), 0.02 mM folic acid (Sigma-Aldrich, USA), and IL-2 (PeproTech, USA), and CAFs were grown in high-glucose Dulbecco's Modified Eagle Medium supplemented with 10% FBS (Gibco, USA). NK-92 cells were used as a reproducible and experimentally tractable NK-cell model for co-culture, metabolic, and genetic manipulation experiments. All cells were incubated at 37 °C in a humidified atmosphere with 5% CO_2_.

### Isolation and culture of CAFs

Fresh osteosarcoma specimens were washed three times with PBS containing 1% penicillin-streptomycin. The tissues were placed in culture dishes with serum-free DMEM/F12 medium containing 1% penicillin-streptomycin. Excess fascia, blood vessels, and necrotic tissue were removed using sterile ophthalmic scissors, and the tissues were minced into 1 mm^3^ fragments. The tissue fragments were evenly spread in culture dishes, and a small amount of DMEM/F12 medium supplemented with 10% FBS and 1% penicillin-streptomycin was added to allow adherence. After 6 h, additional medium was added to completely cover the tissue fragments. The medium was replaced every 3 days until cells grew to confluence. The culture medium was discarded, and cells were washed with PBS, followed by trypsin digestion. After neutralization, cells were resuspended in culture medium and transferred to new dishes. After allowing cells to adhere for 20 min, the culture medium was removed, and the cells were washed with PBS and cultured in fresh medium. After 2-3 passages, purified cancer-associated fibroblasts (CAFs) were obtained using differential adhesion. CAF identity was confirmed by immunofluorescence staining and western blot analysis for α-SMA and FAP. The corresponding representative images and quantitative analysis are shown in Figure S1. Data were obtained from at least three independent experiments and are presented as mean ± SD. Statistical differences between NOFs and CAFs were analyzed using grouped *t*-test. A value of *P* < 0.05 was considered statistically significant.

### Co-culture systems

**NK cell-tumor cell coculture:** NK cells and osteosarcoma cells were cocultured at an effector-to-target (E:T) ratio of 1:1. Briefly, tumor cells were seeded onto sterile coverslips in 24-well plates at a density of 2 × 10^5^ cells per well and allowed to adhere for 6 h at 37 °C in a humidified atmosphere containing 5%CO_2_. Subsequently, NK cells were added at the same density (2 × 10^5^ cells per well) and cocultured for 24 h with direct cell-cell contact. Following coculture, cells and culture supernatants were collected for further analyses.

**CAF-tumor cell coculture:** CAFs and osteosarcoma cells were cocultured at a 1:1 ratio using Transwell inserts (0.4 μm pore size; Corning, NY, USA). Osteosarcoma cells were seeded in the upper chamber at a density of 2 × 10^5^ cells per well, and CAFs were seeded in the lower chamber at the same density. Cocultures were maintained for 48 h at 37 °C in a humidified atmosphere containing 5% CO_2._

**CAF-NK Cell Coculture:** CAFs and NK cells were cocultured using Transwell inserts (0.4 μm pore size; Corning, NY, USA). CAFs were seeded in the upper chamber at a density of 2 × 10^5^ cells per well, and NK cells were seeded in the lower chamber at the same density. NK cells are suspension cells and were seeded in the lower chamber to facilitate direct collection after coculture. Cocultures were maintained for 48 h at 37 °C in a humidified atmosphere containing 5% CO_2_.

The E:T ratios and the concentration of BHB used in this study were selected based on preliminary experimental optimization and previously published *in vitro* studies 29,30. Specifically, 5 mM BHB was used as a literature-supported experimental condition for *in vitro* mechanistic investigation. These standardized conditions were used to improve experimental reproducibility and comparability with previous mechanistic studies.

### Conditioned Medium (CM) preparation

**Exosome-depleted conditioned medium:** CAFs were seeded in 6-well plates. When cells reached 70%-80% confluence, they were treated with the exosome inhibitor GW4869 (Sigma-Aldrich, MO, USA) at a final concentration of 20 μM for 2 h. The medium containing GW4869 was then discarded, and cells were washed twice with PBS, followed by incubation in fresh complete medium for an additional 24 h. The culture supernatant was collected and sequentially centrifuged at 4 °C: 300 × g for 10 min to remove cells and large debris, 3,000 × g for 10 min to remove cell debris and apoptotic bodies, and 10,000 × g for 30 min to remove microvesicles and large particles. The resulting supernatant was filtered through a 0.22 μm filter, aliquoted, and stored at -80 °C until use.

**Protein-depleted conditioned medium:** Exosome-depleted conditioned medium was subjected to three freeze-thaw cycles to disrupt residual vesicles and denature proteins. Each cycle consisted of freezing at -80 °C for 5 min followed by thawing at room temperature for 10 min. After the final thaw, the sample was centrifuged at 14,000 × g for 15 min at 4 °C to remove precipitated proteins and disrupted vesicle fragments. The supernatant was filtered through a 0.22 μm filter, aliquoted, and stored at -80 °C until use.

### Cell counting kit 8 (CCK-8) analysis

NK cell-mediated cytotoxicity toward OS cells was evaluated using an enhanced cell viability assay kit (Procell, P-CA-001). OS cells were seeded into 96-well plates and incubated for 24, 48, or 72 h. Cell counting kit 8 (CCK-8) reagent was then added according to the manufacturer's instructions, followed by incubation at 37 °C for 4 h. Absorbance at 450 nm was measured using a microplate reader.

### Extracellular acidification rate (ECAR) and lactate dehydrogenase activity

NK cells were cultured in medium supplemented with TC supernatants from different treatments. ECAR was assessed using a commercial assay kit (Elabscience, E-BC-F069) by following the manufacturer's instructions, and lactate dehydrogenase activity was subsequently measured.

### Oxygen consumption rate (OCR) assay

NK cells were treated with CAF-conditioned medium and basal and dynamic OCR changes after oligomycin, carbonyl cyanide p-trifluoromethoxy-phenylhydrazone, and rotenone/antimycin A treatment were measured using a Seahorse XFe24 Analyzer according to the manufacturer's instructions for the OCR Fluorometric Assay Kit (Elabscience, E-BC-F068).

### Mitochondrial staining

NK cells were treated with CAF-conditioned medium and subsequently stained with MitoTracker Red CMXRos (Beyotime, C1049B) by following the manufacturer's protocol. Mitochondrial activity in OS cells was visualized using an Olympus laser confocal microscope (Olympus Corporation, Japan).

### Transmission electron microscopy

NK cells treated with CAF-conditioned medium were fixed with 2.5% glutaraldehyde, postfixed with osmium tetroxide, embedded in epoxy resin, and sectioned into ultrathin slices (70 nm). Mitochondrial ultrastructure was examined using a JEM-1400FL transmission electron microscope.

### BODIPY lipid droplet staining

NK cells treated with CAF-conditioned medium were stained with BODIPY™ 493/503 (Thermo Fisher, D3922) according to the manufacturer's instructions. Intracellular lipid droplet distribution was observed using an Olympus confocal microscope (Olympus Corporation, Japan).

### Fatty acid oxidation (FAO) assay

FAO levels in NK cells treated with CAF-conditioned medium were measured using a colorimetric FAO colorimetric assay kit (Elabscience, E-BC-K784-M) according to the manufacturer's instructions.

### Reactive oxygen species (ROS) measurement

ROS levels in NK cells treated with CAF-conditioned medium were assessed using a ROS detection kit (Beyotime, S0034S) according to the manufacturer's protocol, and fluorescence intensity was measured by flow cytometry.

### Cell transfection

One day prior to transfection, NK92 cells were seeded in 24-well plates at a density of 2 × 10^5^ cells per well in 500 µL of complete medium without antibiotics. Transfection was performed using GLP Lipo3000 Transfection Reagent (GlpBio, GK20006) according to the manufacturer's instructions.

**For plasmid DNA transfection:** For each well, 1 µg of plasmid DNA and 1 µL of P3000 reagent were diluted in 25 µL of Opti-MEM I reduced serum medium (Gibco, 31985062). Separately, 1.5 µL of Lipo3000 Transfection Reagent was diluted in 25 µL of Opti-MEM I medium. The diluted DNA mixture was combined with the diluted Lipo3000 reagent and gently mixed. The mixture was incubated at room temperature for 10-15 min to allow complex formation. Subsequently, 50 µL of the transfection complex was added dropwise to the cells. The plate was gently swirled to ensure uniform distribution. Cells were incubated at 37 °C with 5% CO_2_ for 48 h prior to subsequent experiments. Plasmid sequences details are listed in Table 1.

**For siRNA transfection:** For each well, 15 pmol of siRNA was diluted in 25 µL of Opti-MEM I medium. Separately, 1.5 µL of Lipo3000 Transfection Reagent was diluted in 25 µL of Opti-MEM I medium. The diluted siRNA was mixed with the diluted Lipo3000 reagent and gently mixed. The mixture was incubated at room temperature for 10-15 min. Then, 50 µL of the transfection complex was added dropwise to the cells, followed by gentle mixing. Cells were cultured for 72 h at 37 °C with 5%CO_2_ before further analysis. siRNA sequences are listed in Table 2.

### RNA extraction and reverse transcription quantitative polymerase chain reaction (RT-qPCR)

Total RNA was extracted using TRIzol reagent (Thermo Fisher, 15596018CN). Complementary DNA synthesis was performed using the PrimeScript™ reverse transcription kit (TAKARA, RR037A) in a 20 μL reaction containing 1 μg RNA, 4 μL 5× buffer, and 1 μL reverse transcriptase. Quantitative polymerase chain reaction (qPCR) was performed using TB Green® Premix Ex Taq™ II (TAKARA, RR820A) on a CFX96 system (Bio-Rad) under the following conditions: 95 °C for 30 s, 40 cycles of 95 °C for 5 s, and 60 °C for 30 s. RT-qPCR primer sequences are listed in Table 3.

### Western blot analysis

Cells cultured in six-well plates were harvested, lysed on ice with cell lysis buffer, and sonicated. After centrifugation at high speed and low temperature, supernatants were collected, mixed with a 250 μL loading buffer, and denatured to prepare the protein samples. Proteins were separated by 12.5% sodium dodecyl sulfate-polyacrylamide gel electrophoresis and transferred onto polyvinylidene difluoride membranes. Membranes were blocked with 5% nonfat milk and incubated with primary antibodies overnight at 4 °C, followed by corresponding secondary antibodies (MultiSciences, GAM0072 and GAR007). Protein bands were visualized using an enhanced chemiluminescence kit (Beyotime, P0018S).

### Co-Immunoprecipitation (Co-IP)

NK92 cells treated according to the experimental design were lysed in NP-40 lysis buffer (Beyotime, P0013F) supplemented with protease inhibitor cocktail and phenylmethylsulfonyl fluoride (PMSF). Cell lysates were centrifuged at 12,000 × g for 15 min at 4 °C to remove insoluble debris. The supernatants were collected and incubated with protein A/G magnetic beads conjugated with antibodies against Kbhb, FXR1, or Flag overnight at 4 °C with gentle rotation. After incubation, the beads were washed five times with IP washing buffer to remove non-specifically bound proteins. Bound proteins were eluted by boiling the beads in SDS-PAGE loading buffer and subjected to Western blot analysis.

### Cellular immunofluorescence

Cells cultured on coverslips in 24-well plates were fixed with 4% paraformaldehyde for 20 min, permeabilized with 0.5% Triton X-100 for 15 min, and blocked with 5% bovine serum albumin for 1 h. Primary antibodies were incubated overnight at 4 °C, followed by secondary antibodies for 1 h in the dark. Nuclei were counterstained with 4′,6-diamidino-2-phenylindole (Beyotime, C1006) for 15 min. Images were captured using an Olympus laser confocal microscope.

**Primary antibodies:** Human leukocyte antigen class I histocompatibility antigen, alpha chain E (HLA-E; Proteintech, CL488-66530, 1:200), natural killer group 2 member C (NKG2C; Abcam, ab220898, 1:200), RNA-binding protein FXR1 (FXR1; Proteintech, 13194-1-AP, 1:200), histone acetyltransferase p300 (P300; Cell Signaling Technology, 86377S, 1:400), NAD-dependent protein deacetylase sirtuin-2 (SIRT2; Proteintech, 19655-1-AP, 1:200).

**Secondary antibodies:** Cy3-conjugated goat antimouse immunoglobulin G (IgG) (H+L) (Proteintech, SA00009-1, 1:50), Cy3-conjugated goat antirabbit IgG (H+L) (Proteintech, SA00009-2, 1:100), fluorescein isothiocyanate (FITC)-conjugated goat antimouse IgG (H+L) (Proteintech, SA00003-1, 1:100), FITC-conjugated goat antirabbit IgG (H+L) (Proteintech, SA00003-2, 1:100).

### Flow cytometry

Cocultured cells were collected and incubated with anti-CD16/32 to block fragment crystallizable receptors, followed by staining with FITC-CD314 antibody (Proteintech, FITC-65643, 5 μL/10⁶ cells) for 30 min at 4 °C in the dark. Cells were washed with PBS, and CD314 expression was analyzed using BD FACSDiva software.

### Chromatin immunoprecipitation (ChIP)

ChIP was performed using the SimpleChIP® Plus Sonication ChIP Kit (Cell Signaling Technology, 56383S). Cells were fixed with formaldehyde and then lysed, and chromatin was sonicated to ~0.5 kb fragments. Immunoprecipitation (IP) was performed with anti-H3K9bhb antibody (PTM Bio, PTM-1250) overnight at 4 °C. ChIP-grade protein G magnetic beads were added and rotated at 4 °C for 2 h. Beads were washed, and DNA was eluted and purified for the qPCR analysis of promoter region modifications.

### Animal models and CAF-CM treatment

Animal experiments were performed in strict accordance with the recommendations of the Guide for the Care and Use of Laboratory Animals of the National Institutes of Health. All experimental protocols were approved by the Animal Experimental Ethics Committee of Huazhong University of Science and Technology ([2025] IACUC Number: 4807). Male BALB/c nude mice (three weeks old, specific pathogen-free grade) were subcutaneously injected with 5 × 10⁶ 143B cells in the right femur. Mice were randomized into the PBS or CAF-conditioned medium treatment group. The xenograft model combined human 143B osteosarcoma cells and human CAF-derived conditioned medium with BALB/c nude mice. Therefore, the intratumoral NK-cell-related readouts reflected host murine NK cells rather than the human NK-92 cells used in the *in vitro* experiments. From day 7 postinoculation, 75 μL of PBS or CAF-conditioned medium was injected peritumorally every two days for a total of five injections. Tumor dimensions (length, L; width, W) were measured every three days, and volume was calculated as V = 0.5 × L × W². Mice were euthanized on day 21 with 10% chloral hydrate. Serum β-hydroxybutyrate (BHB) levels were measured using a colorimetric assay kit (Abcam, ab83390). Tumor tissues were processed into single-cell suspensions for flow cytometric analysis of CD314 expression or embedded in optimal cutting temperature compound for Nile red staining (MedChemExpress, HY-D0718, 100 ng/mL, 10 min) and fluorescence microscopy of lipid droplets.

### Silver staining

Protein β-hydroxybutyrylation (Kbhb) in NK cells treated with CAF supernatant or BHB was detected using a rapid silver staining kit (Beyotime, P0017S) by following the manufacturer's instructions. Kbhb-modified proteins were enriched using a Kbhb-specific antibody.

### Mass spectrometry

Protein lysates from NK92 cells treated with CAF-CM or BHB were collected, and β-hydroxybutyrylated proteins were enriched using a Kbhb antibody. The enriched proteins were sequentially processed as follows: heat denaturation at 95 °C for 10 min, trichloroacetic acid (TCA) precipitation, reduction and alkylation at 60 °C for 30 min, trypsin digestion at 37 °C overnight, termination with trifluoroacetic acid (TFA), centrifugation, and desalting using SDB columns. The resulting peptides were lyophilized and stored for subsequent analysis.

The lyophilized peptides were analyzed using a nano-liquid chromatography system (UltiMate 3000 RSLCnano) coupled to a high-resolution Q Exactive HF mass spectrometer. Peptides were separated on a C18 trap column and an analytical column using a formic acid-acetonitrile gradient containing DMSO. The mass spectrometer was operated in data-dependent acquisition mode. Full MS scans were acquired at a resolution of 60,000 over a mass range of 350-1500 m/z. The top 20 most intense precursor ions were selected for fragmentation using higher-energy collisional dissociation (HCD) with a normalized collision energy of 28%. MS/MS spectra were acquired at a resolution of 15,000, and dynamic exclusion was set to 30 s.

### Statistical analysis

All experiments were performed at least three times. Data are presented as mean ± standard deviation. Statistical analyses were conducted using GraphPad Prism 8.0 (GraphPad Software, San Diego, CA, USA). For comparisons among three or more groups, one-way or two-way analysis of variance (ANOVA) was used as appropriate, followed by Tukey's post hoc test for multiple comparisons or Dunnett's post hoc test for comparisons against a single control group. Data normality was assessed using the Shapiro-Wilk test prior to parametric analyses. P < 0.05 was considered statistically significant (*P < 0.05, **P < 0.01, ***P < 0.001).

## Results

### CAFs suppress NK-cell activation and cytotoxicity against OS cells

To evaluate the ability of NK cells to recognize and kill OS cells, we first established an NK cell-tumor cell coculture system (i.e., NK-TC) at different effector-to-target ratios (Fig. 1A). CCK-8 assays showed that NK cells exerted a marked cytotoxic effect when cocultured with OS cells at a 1:1 ratio (Fig. 1B). Meanwhile, ELISA analysis showed that the secretion levels of IFN-γ and perforin were significantly upregulated after coculture of NK cells with osteosarcoma cells (Fig. 1C-D). HLA-E, which is also referred to as major histocompatibility complex (MHC) class I antigen E, plays a pivotal role in NK-cell recognition. NKG2C is an activating receptor expressed on the surface of NK cells that forms a heterodimer with CD94 and specifically interacts with HLA-E, thereby promoting NK cell cytotoxicity and cytokine production 31-33. Immunofluorescence staining further demonstrated the colocalization of HLA-E on tumor cells with NKG2C on NK cells, thus confirming their direct interaction (Fig. 1E). Consistently, flow cytometry analysis revealed NK-cell activation rates of 19.5% and 40.2% under these conditions (Fig. 1F).

To investigate whether CAFs influence NK-cell activity, we generated a CAF-TC coculture system and added CAF-conditioned medium (CAF-CM) from this culture into the NK-TC cocultures (Fig. 1G). CCK-8 assays demonstrated that the addition of CAF-CM promoted OS-cell proliferation in a CAF dose-dependent manner while attenuating NK cell-mediated cytotoxicity (Fig. 1H). Immunofluorescence analysis showed the reduced colocalization of HLA-E and NKG2C in the presence of CAF-CM, thus indicating the impaired recognition of tumor cells by NK cells (Fig. 1I). CD314 (also known as NKG2D), which is encoded by the *KLRK1* gene, is a representative activating receptor expressed on human and murine NK cells, CD8⁺ T cells, and other immune effector cells 34-36. CD314 recognizes MHC class I polypeptide-related sequence A/B and UL16-binding protein family ligands to trigger cytotoxicity and cytokine release 37. Flow cytometry further confirmed that NK-cell activation was suppressed, with activation rates reduced to 10.42% and 9.96% (Fig. 1J).

These results indicate that CAFs suppress NK-cell recognition and activation, thereby impairing NK-cell cytotoxicity against OS cells.

### CAFs induce the metabolic reprogramming of NK cells to promote tumor immune suppression

Studies have shown that CAFs undergo metabolic reprogramming to generate amino acids and other nutrients that sustain tumor growth and invasion; furthermore, they can secrete metabolites such as lactate, amino acids, and lysophosphatidylcholine into the TME to alter the metabolism of neighboring cells 38. To determine whether CAFs regulate NK-cell metabolism, we treated NK cells cocultured with OS cells by using CAF-CM and assessed glycolytic and mitochondrial activity by using ECAR and OCR assays. CAF-CM significantly increased ECAR while reducing OCR, with OCR levels remaining relatively stable over time (Fig. 2A-2B). These findings indicate that CAFs shift NK cells toward glycolytic metabolism with reduced mitochondrial respiration.

ROS measurements revealed that CAF-conditioned NK cells exhibited elevated oxidative stress, with intracellular ROS levels increased by 24.9% and 19.1% (Fig. 2C); this finding is consistent with reports that lactate-driven glycolysis activates ROS signaling 39. MitoTracker staining further demonstrated reduced mitochondrial activity, with fluorescence intensity decreasing by approximately two-thirds and one-half in NK cells treated with supernatants from two OS-derived CAF cocultures (Fig. 2D). Electron microscopy confirmed these findings by showing swollen and damaged mitochondrial membranes with disrupted cristae in CAF-conditioned NK cells (Fig. 2E). These data demonstrate that CAFs impair NK-cell energy metabolism by inducing mitochondrial structural and functional abnormalities.

To determine whether tumor cells themselves contributed to NK-cell metabolic alterations, we compared the effects of tumor-CM and tumor/CAF-CM on NK cells. Compared to the tumor-CM group, the tumor/CAF-CM group induced significant alterations in ECAR and OCR in NK cells (Fig. 2F-2G). These results suggest that CAFs may indirectly regulate NK-cell metabolic reprogramming in the OS TME through modulation of tumor cells.

Finally, immunofluorescence analysis showed that CAF-CM impaired NK-cell recognition of OS cells, as evidenced by the reduced colocalization of HLA-E and NKG2C, thus leading to diminished NK-cell cytotoxicity (Fig. 2H).

### CAFs suppress fatty acid uptake and oxidation in NK cells

Lipid metabolism is essential for sustaining NK-cell effector functions 40. Building on our earlier findings that CAFs reprogram NK-cell metabolism to impair antitumor activity, we investigated their effects on fatty acid metabolism in NK cells.

BODIPY lipid droplet staining revealed a marked reduction in intracellular lipid content in NK cells exposed to CAFs, showing more than a twofold decrease compared with controls (Fig. 3A). Consistently, FAO assays demonstrated significantly reduced FAO levels in NK cells treated with CAFs (Fig. 3B), thus suggesting that CAFs impair both lipid acquisition and oxidation capacity.

Molecular analyses supported these observations: RT-qPCR and western blotting showed that the expression of lipid oxidation-related genes, including *SREBP* and *PPARG*, was markedly downregulated in NK cells cultured with CAF-CM (Fig. 3C-3D). These findings establish that CAFs profoundly suppress NK-cell FAO activity.

To determine whether this suppression resulted from direct metabolic competition or paracrine signaling, we first cultured CAFs and NK cells separately and assessed lipid uptake at 0, 12, and 24 h in the presence of fluorescently labeled lipids (Fig. 3E). The results showed that both CAFs and NK cells exhibited strong lipid uptake when cultured independently, which increased over time. Subsequently, we treated NK cells with CAF-CM and assessed lipid uptake using BODIPY fluorescence labeling (Fig. 3F). The results showed that NK cell lipid uptake was significantly inhibited after CAF-CM treatment (Fig. 3G), indicating that CAFs suppress NK cell lipid uptake through paracrine signaling.

To investigate the critical role of fatty acid oxidation in CAF-induced NK cell dysfunction, we performed rescue experiments by treating NK cells with the PPARα agonist GW7647 to activate fatty acid oxidation in the presence of CAF-CM. The results showed that GW7647 treatment significantly rescued the inhibitory effect of CAF-CM on NK cell recognition, as evidenced by enhanced colocalization of HLA-E and NKG2C (Fig. 3H). Meanwhile, GW7647 treatment also significantly restored IFN-γ secretion in NK cells (Fig. 3I). These findings indicate that impaired fatty acid oxidation is a key mechanism underlying CAF-mediated NK cell dysfunction.

Collectively, these data demonstrate that CAFs impair NK-cell lipid uptake and FAO primarily through secreted factors, thereby contributing to NK-cell metabolic dysfunction within the OS microenvironment.

### CAF-derived metabolite BHB inhibits NK cell lipid metabolism and tumor recognition

Studies confirmed that CAFs can modulate NK-cell lipid metabolism through their secreted factors. However, the specific component responsible for this effect remained unclear. Prior studies have shown that intercellular communication is generally mediated by three main mechanisms: direct cell-cell contact, secretion of soluble factors, and ECM remodeling, which collectively influence the surrounding microenvironment and neighboring cells 41-43. Among these, CAFs can communicate with other cells via secreted metabolites or extracellular vesicles (EVs) 44. To identify the CAF-derived components that impair NK-cell lipid metabolism and mediate immunosuppression, we prepared CAF-CM, CAF-derived EVs, CAF-CM treated with the exosome inhibitor GW4869, and CAF-CM subjected to three freeze-thaw cycles following GW4869 treatment. Each component, as well as PBS as a control, was added to NK-TC coculture systems (schematic shown in Fig. 4A).

NK-cell recognition of tumor cells was subsequently assessed. Immunofluorescence analysis revealed strong HLA-E/NKG2C signals in the PBS and CAF-EV groups, whereas NK cells treated with any form of CAF-CM, including GW4869-treated or freeze-thawed CM, showed markedly reduced tumor recognition compared to the PBS or CAF-EV groups (Fig. 4B). These results indicate that CAF-derived EVs contribute minimally to blocking NK-cell tumor recognition, whereas CAF metabolites in the CM are key mediators of NK-cell immunosuppression. Given that CAF metabolites can modulate NK-cell tumor recognition and that CAFs exhibit enhanced lipid metabolism, we hypothesized that lipid-derived metabolites are likely responsible for influencing NK-cell activity.

To test this hypothesis, common CAF lipid metabolites, acetyl coenzyme A, BHB, and lactate, were individually added to NK-TC cocultures to evaluate their effects on NK-cell tumor recognition and activation. Acetyl coenzyme A, BHB, and lactate were individually added to NK-TC cocultures at final concentrations of **100 μM**, **5 mM**, and **10 mM**, respectively 29,45-48. These concentrations were selected based on previous studies of the corresponding metabolites. Flow cytometry analysis revealed that NK cells treated with BHB exhibited significantly lower CD314 expression than the other treatment groups (Fig. 4D), thus indicating reduced NK-cell activation. The colocalization analysis of HLA-E/NKG2C immunofluorescence signals further demonstrated that, relative to acetyl coenzyme A and lactate, BHB treatment markedly suppressed NK-cell tumor recognition, as indicated by weaker fluorescence intensity (Fig. 4C).

Given that BHB was identified as a key metabolite in CAF-CM mediating NK cell immunosuppression, we further investigated whether CAFs serve as the primary source of BHB in the osteosarcoma tumor microenvironment. First, we measured the basal BHB secretion levels in NK cells, tumor cells, and CAFs. The results showed that CAFs exhibited significantly higher basal BHB secretion capacity than tumor cells and NK cells (Fig. S2A). Further analysis revealed that after co-culture with tumor cells, BHB secretion levels in CAFs were markedly upregulated, accompanied by significantly increased expression of the ketogenic key enzymes HMGCS2 and BDH1 (Fig. S2B-S2C). To validate the regulatory role of CAF-derived BHB on NK cell function, we knocked down HMGCS2 expression in CAFs following co-culture with osteosarcoma cells, which effectively abrogated BHB production in CAFs (Fig. S2D). Subsequently, NK cells were treated with the conditioned medium from these HMGCS2-knockdown CAFs; the results showed that fatty acid oxidation levels and lipid uptake capacity were significantly increased (Fig. S2E-S2F), and the secretion level of IFN-γ was also markedly upregulated (Fig. S2G). These findings support CAF-derived BHB as an important contributor to the metabolic and functional suppression of NK cells *in vitro*, while not excluding the involvement of other CAF-secreted factors.

BHB, a major ketone body, can serve as an alternative energy source in certain tissues 49. Accumulating evidence indicates that BHB promotes tumor progression in multiple cancer types, including pancreatic, colorectal, and melanoma 50-52. Thus, we investigated whether BHB modulates NK-cell lipid metabolism and immune function. FAO analysis revealed a significant reduction in FAO in NK cells treated with BHB (Fig. 4E). Consistently, RT-qPCR and western blot assays showed that BHB treatment significantly suppressed the expression of FAO-related genes *SREBP* and *PPARG* (Fig. 4F-4G). Moreover, CCK-8 assays confirmed that BHB effectively inhibited NK cell-mediated cytotoxicity against tumor cells (Fig. 4H). Meanwhile, ELISA assays showed that BHB treatment reduced IFN-γ and perforin secretion, consistent with impaired NK-cell effector function (Fig. 4I-4J).

Together, these data show that BHB contributes to reduced NK-cell FAO and immunosuppressive function *in vitro*.

### CAF-CM treatment is accompanied by elevated circulating BHB and impaired NK-cell activity *in vivo*

To determine whether CAF-CM treatment was accompanied by coordinated changes in circulating BHB levels and NK-cell function *in vivo*, we established a subcutaneous osteosarcoma xenograft model (Fig. 5A). Beginning on day 7 after tumor implantation, mice received peritumoral injections of 75 μL PBS or CAF-CM every other day, and tumor volumes were measured at regular intervals. On day 21, tumors were excised for gross examination. Tumor volumes were significantly larger in the CAF-CM group compared with the controls (Fig. 5B).

Growth curve analysis based on serial measurements revealed accelerated tumor progression in the CAF-CM-treated mice, with a markedly faster growth rate after day 12 and larger terminal tumor volumes than those in the control group (Fig. 5C).

Consistently, systemic BHB levels were significantly elevated in CAF-CM-treated mice compared with the controls (Fig. 5D). Flow cytometric analysis showed a reduced proportion of CD314-positive cells in tumors from CAF-CM-treated mice (Fig. 5E). Histological examination with Nile red staining revealed strong orange-red fluorescence corresponding to lipid droplets in control tumors, with a high degree of colocalization with NKG2C⁺ NK cells. By contrast, CAF-CM-treated tumors exhibited weaker lipid signals and poor overlap with NKG2C, thus indicating reduced lipid accumulation in NK cells (Fig. 5F). Meanwhile, Western blotting of whole-tumor lysates showed reduced Granzyme A and perforin expression in the CAF-CM-treated group (Fig. 5G). These results are consistent with our *in vitro* findings, where CAF-CM decreased FAO levels and impaired NK cytotoxicity.

To further investigate the role of CAF-CM in tumor development and progression, we established an orthotopic tibial injection model to recapitulate the bone microenvironment of osteosarcoma. The results showed that tumor volume in the tibial region was significantly larger in the CAF-CM-treated group compared with the control group (Fig. 5H). Micro-computed tomography (micro-CT) analysis showed that trabecular bone mass in the proximal tibia was significantly reduced in the CAF-CM-treated group (Fig. 5I). Quantitative analysis demonstrated that bone volume fraction (BV/TV), trabecular thickness (Tb.Th), and trabecular number (Tb.N) were all significantly decreased, whereas trabecular separation (Tb.Sp) showed no significant change (Fig. 5J).

Overall, CAF-CM treatment was accompanied by accelerated tumor growth, elevated circulating BHB, and reduced NK-cell-associated activation and lipid readouts, thereby establishing an immunosuppressive TME.

### CAF-derived BHB suppresses NK-cell FAO via P300-mediated Kbhb of FXR1

Studies have demonstrated that BHB serves not only as a metabolic substrate maintaining energy homeostasis but also as an epigenetic regulator, modulating disease processes such as metabolic syndrome, cancer, and neurodegeneration through mechanisms including histone methylation, acetylation, and Kbhb 53. To elucidate how CAF-derived BHB influences NK-cell lipid uptake and modulates immune function, we investigated its effect on protein Kbhb in NK cells. Kbhb is a novel lysine acylation modification mediated by BHB and was first reported by Zhao *et al*. in 2016; it is closely associated with FAO and energy metabolism regulation 54.

We first assessed Kbhb levels in NK cells following BHB treatment or exposure to CAF-CM. IP with two independent Kbhb antibodies confirmed enrichment of Kbhb-modified proteins (Fig. 6A). Western blotting further validated that both BHB and CAF-CM enhanced global Kbhb levels in NK cells (Fig. 6B). To explore subcellular distribution, cytoplasmic, nuclear, and mitochondrial fractions were analyzed. The results revealed that Kbhb modification was markedly higher in nuclear and mitochondrial proteins than in cytoplasmic proteins (Fig. 6C).

To identify the potential mediators of CAF-induced metabolic regulation, Kbhb-enriched proteins from BHB- or CAF-CM-treated NK cells were subjected to mass spectrometry (Fig. 6D). Among eight overlapping proteins, two were associated with FAO: transferrin and RNA-binding protein FXR1. Western blotting of Kbhb-immunoprecipitated products confirmed FXR1 as the predominant target (Fig. 6E), thus implicating FXR1 as a key mediator of CAF-CM BHB-induced Kbhb modification in NK cells.

FXR1, an RNA-binding protein, regulates mRNA translation and stability and participates in diverse biological processes 55-57. To examine how Kbhb affects FXR1 function, NK cells were transfected with an FXR1-overexpression plasmid. Transfection efficiency was confirmed by qPCR and western blotting (Figs. 6F-6G). Functional assays demonstrated that FXR1 overexpression significantly enhanced NK cell FAO (Fig. 6H), whereas reverse IP revealed elevated Kbhb modification of FXR1 upon BHB or CAF-CM treatment (Fig. 6I), thus confirming successful overexpression and posttranslational modification.

To elucidate the enzymatic mechanism responsible for FXR1 Kbhb modification, we focused on P300, which has been well characterized as an acyltransferase that catalyzes acetylation, propionylation, and butyrylation of lysine residues 58-60. Emerging evidence has revealed that P300 also possesses the capacity to mediate lysine Kbhb 54,61. Immunofluorescence analysis showed enhanced cytoplasmic colocalization of FXR1 with P300 after BHB or CAF-CM treatment (Fig. 6J). Consistently, IP-western blotting confirmed an increased interaction between FXR1 and P300 under these conditions (Fig. 6K). P300 knockdown efficiency was subsequently confirmed at both the mRNA and protein levels (Fig. 6L-6M). Notably, P300 knockdown significantly increased FAO activity in NK cells compared with the si-NC group (Fig. 6N), consistent with an inhibitory role of P300 in the regulation of NK-cell FAO. Given the broad role of P300 as a transcriptional coactivator and acyltransferase, this result should be interpreted with caution, as si-P300-mediated FAO increase may reflect the global metabolic consequence of P300 depletion rather than a specific reversal of BHB-induced FAO suppression.

Together, the enhanced FXR1-P300 colocalization and interaction following BHB or CAF-CM exposure, combined with the increase in FAO after P300 knockdown, support the involvement of P300 in BHB-associated FXR1 Kbhb and NK-cell FAO suppression. These findings are consistent with a model in which P300-mediated FXR1 Kbhb contributes to impaired FAO in NK cells. Nevertheless, because the current FAO experiment did not directly compare BHB-treated si-NC and si-P300 cells, further catalytic-loss-of-function or rescue experiments will be required to establish the specific enzymatic dependence of this process.

### BHB induces a shift from acetylation to Kbhb at FXR1 K56

To further identify the specific Kbhb modification site on FXR1, we used the pFunK online tool (http://pfunk.biocuckoo.cn/online.php) to predict potential lysine residues that are susceptible to Kbhb. Three candidate sites were identified: K56, K273, and K310 (Fig. 7A).

To determine which site undergoes Kbhb modification, FXR1 was divided according to its domain structure into full length and residues 1-120, 121-282, and 283-621 (Fig. 7B). Corresponding FLAG-tagged FXR1 constructs were expressed in NK cells, and IP-western blotting was performed (Fig. 7C). Kbhb levels were assessed for each fragment, revealing that the 1-120 region, encompassing K56, exhibited the strongest enrichment of Kbhb modification (Fig. 7D), indicating that K56 represents the core site of FXR1 Kbhb modification.

Subsequently, a K56 mutant FXR1 construct was generated (Fig. 7E). Immunofluorescence analysis demonstrated that the K56 mutation markedly reduced the colocalization signal between FXR1 and P300, accompanied by increased nuclear localization of FXR1 (Fig. 7F). Consistently, IP-western blot experiments confirmed that the interaction between FXR1 and P300 was significantly diminished in the K56 mutant (Fig. 7G), thus indicating that K56 is critical for FXR1-P300 binding.

Further analysis revealed that K56 is a potential site for multiple posttranslational modifications, including acetylation, SUMOylation, and ubiquitination (Fig. 7H). Western blot analysis showed that BHB treatment markedly decreased FXR1 acetylation, whereas SUMOylation and ubiquitination were minimally affected (Fig. 7I), thus suggesting that K56 is a key residue subject to multiple modifications. Notably, compared with wild-type K56, BHB treatment increased the acetylation level of K56 mutant FXR1 (Fig. 7J), thus indicating that K56 is a critical regulatory site mediating the switch from acetylation to Kbhb under BHB exposure.

To examine the contribution of FXR1 K56 modification to BHB-associated NK-cell dysfunction, we performed rescue experiments using the FXR1-K56R mutant in NK cells and assessed the expression levels of IFN-γ and perforin. The results showed that K56R mutation significantly reversed the downregulation of IFN-γ and perforin expression induced by BHB treatment (Fig. 7K-7L), indicating that BHB mediates its inhibitory effect on NK cell function through the induction of FXR1-K56 Kbhb modification.

### BHB-mediated H3K9bhb modification activates SIRT2 to promote FXR1 deacetylation

Collectively, our data indicate that FXR1 deacetylation is a critical step for its functional suppression and subsequent conversion to Kbhb modification. Histone deacetylases (HDACs) and sirtuins (SIRTs) are key regulators of deacetylation processes and play pivotal roles in controlling chromatin structure and gene expression 62,63. HDACs remove acetyl groups from lysine residues on histones, thus promoting tighter interaction with negatively charged DNA and influencing chromatin remodeling and transcriptional regulation 64. SIRTs are evolutionarily conserved NAD⁺-dependent deacetylases involved in cellular metabolism, mitochondrial dynamics, and oxidative stress resistance 65-67. Notably, BHB can modulate signaling pathways and inhibit nuclear HDACs, thus suggesting a potential link between BHB and HDACs/SIRTs in NK cells 68,69.

To investigate whether BHB affects HDAC and SIRT expression, we analyzed NK cells treated with BHB or CAF-CM and measured the expression levels of both enzyme families. Intersection analysis revealed two shared targets: HDAC5 and SIRT2 (Fig. 8A).

We then evaluated the interaction of HDAC5 and SIRT2 with FXR1 via immunoprecipitation. The results indicated that SIRT2 exhibited a stronger binding affinity to FXR1 than HDAC5 (Fig. 8B), prompting further mechanistic studies focused on SIRT2. IP assays demonstrated that BHB or CAF-CM treatment enhanced the interaction between SIRT2 and FXR1 in NK cells (Fig. 8C). Immunofluorescence further confirmed the increased colocalization of SIRT2 and FXR1 following BHB or CAF-CM exposure, thus supporting the notion that BHB promotes SIRT2-FXR1 complex formation (Fig. 8D).

SIRT2 overexpression increased FXR1 Kbhb levels, supporting the potential involvement of SIRT2 in FXR1 modification (Fig. 8E). By using data from the genome browser of the University of California, Santa Cruz, we identified active regions in the SIRT2 promoter and designed ChIP-qPCR primers accordingly (Fig. 8F). ChIP-qPCR analysis revealed increased H3K9bhb enrichment at the SIRT2 promoter after BHB or CAF-CM treatment, consistent with the observed increase in SIRT2 expression (Fig. 8G).

Together, these findings suggest that BHB or CAF-CM treatment is associated with SIRT2 upregulation and enhanced SIRT2-FXR1 interaction.

## Discussion

This study found a novel mechanism by which CAFs in the TME suppress NK-cell effector functions through metabolic reprogramming. Specifically, CAF-derived BHB impairs NK-cell FAO, reduces mitochondrial membrane potential, and diminishes secretion of cytotoxic molecules such as IFN-γ and granzyme B, thus concurrently inhibiting lipid uptake. These findings reveal that CAFs extend beyond the structural and cytokine-mediated modulation of the immune environment, thus directly influencing immune cell metabolism.

Metabolic byproducts have emerged as key modulators of the immune landscape in solid tumors 70. For example, lactate and adenosine accumulate in the TME and suppress effector immune cells via acidification or signaling pathways, thus facilitating immune escape 71,72. This focus does not exclude the contribution of other classical immunosuppressive metabolites, but rather highlights an underexplored layer of CAF-mediated immune regulation. Unlike lactate or adenosine, which mainly act through metabolic stress, acidification, or receptor-mediated signaling, BHB may additionally influence immune-cell function through lysine β-hydroxybutyrylation-dependent epigenetic regulation. Therefore, BHB provides a mechanistic bridge between CAF metabolic remodeling and NK-cell lipid metabolic dysfunction. Recent studies have revealed that BHB functions as an epigenetic modulator to regulate immune cell function. For instance, Luo *et al*. demonstrated that BHB induces Kbhb modification of STAT1 at lysine 679, inhibiting its phosphorylation and transcriptional activity and thereby modulating macrophage polarization 73. Hao *et al*. reported that BHB promotes Treg cell differentiation by enhancing histone H3 acetylation at the Foxp3 locus 74. Consistent with these findings, we discovered that within the OS TME, BHB deacetylates the RNA-binding protein FXR1, thus blocking NK-cell FAO and limiting metabolic flexibility and cytotoxicity. BHB exposure induces coordinated and consistent metabolic and functional changes in NK cells, including impaired lipid metabolism, reduced mitochondrial activity, and diminished antitumor function. This metabolic-epigenetic coupling provides a mechanistic explanation for NK-cell dysfunction in solid tumors. Importantly, lipid metabolism is essential for NK-cell longevity and memory-like differentiation. Our findings directly link this metabolic dependency to CAF-derived metabolites, thus emphasizing that TME-driven metabolic remodeling is a fundamental determinant of NK-cell effector function.

CAFs have long been implicated as drivers of tumor growth and immune suppression through the secretion of cytokines such as interleukin 6 and transforming growth factor beta and the deposition of ECM components 19. The results of the current study extend this paradigm by highlighting the metabolic contribution of CAFs: CAFs derived from OS secrete high levels of BHB, which markedly diminishes NK-cell lipid utilization and effector secretion in coculture systems. This dual role positions CAFs as central regulators within the tumor ecosystem, orchestrating both metabolic reprogramming and immune suppression. While our data support CAFs as a major source of immunosuppressive BHB in this context, we acknowledge that within the complex and heterogeneous TME, other cell types, including tumor cells themselves under certain stress conditions, could also contribute to the local BHB pool. A systematic comparison of BHB production capacity across different cellular components of the TME will be important to fully delineate the source and dynamics of this metabolite. Whether intratumoral or circulating BHB levels are associated with chemotherapy response or prognosis in osteosarcoma requires validation in clinically annotated cohorts.

The direct BHB-treatment experiments and the partial restoration of NK-cell FAO, lipid uptake, and IFN-γ secretion after HMGCS2 knockdown in CAFs support BHB as an important contributor to CAF-mediated NK-cell dysfunction *in vitro*. *In vivo*, CAF-CM treatment was accompanied by elevated circulating BHB and coordinated impairment of NK-cell activation, lipid accumulation, and cytotoxic molecule expression. However, these findings do not identify BHB as the sole mediator of the CAF-CM effect, nor do they establish that accelerated tumor growth is dependent on NK cells. BHB-specific intervention and NK-cell depletion studies will be required to resolve these questions.

Osteosarcoma is a highly heterogeneous disease comprising multiple histopathological subtypes that exhibit distinct clinical behaviors, treatment responses, and tumor microenvironment compositions 75. The cell lines used in this study (143B and KHOS) are commonly employed to model conventional central osteosarcoma, particularly the osteoblastic and fibroblastic subtypes. Therefore, whether the CAF-BHB-NK cell immunosuppressive axis identified here represents a general mechanism across all osteosarcoma subtypes or exhibits subtype-specific features remains to be determined and warrants further investigation. Similarly, it is unclear whether BHB exerts differential immunomodulatory effects across these varied microenvironments, an important question for future investigation. Notably, cancer-associated fibroblasts themselves exhibit significant functional heterogeneity, with distinct subtypes displaying different metabolic and immunomodulatory properties 76,77. The CAFs used in this study were characterized by positive expression of α-SMA and FAP, confirming their identity as activated CAFs. Given that myofibroblastic CAFs (myCAFs) are metabolically active and have been implicated in metabolic reprogramming within the osteosarcoma microenvironment 78, the robust BHB production observed in our study likely originates from this CAF subset. However, whether BHB production is a general feature of all activated CAFs or is restricted to specific subpopulations warrants further clarification. Emerging single-cell transcriptomic approaches represent a promising strategy to deconvolve CAF heterogeneity and precisely map the BHB-producing capacity to specific stromal subsets in future work. Future studies incorporating subtype-stratified models and single-cell transcriptomic analyses will be critical to elucidate the heterogeneity of BHB-mediated immunoregulation across osteosarcoma subtypes.

Beyond the cellular source and context, our data demonstrate that BHB simultaneously triggers impaired fatty acid oxidation, mitochondrial dysfunction, and reduced cytotoxicity. While the consistency of these events suggests FAO impairment is a key component of this regulatory process, the precise causal hierarchy warrants further investigation. It remains to be determined whether suppressed FAO is the primary driver of downstream mitochondrial and functional defects, or a consequence of a broader BHB-induced metabolic disruption. Future studies employing targeted metabolic rescue experiments, such as providing exogenous fatty acids, will be essential to clarify this regulatory network.

The identified CAF-BHB-FXR1-NK cell axis represents a promising therapeutic target. Interventions that inhibit BHB production or disrupt downstream signaling may restore NK-cell metabolism and cytotoxicity, thus enhancing TC clearance. Moreover, metabolic modulation strategies may complement immune checkpoint inhibitors to improve immunotherapeutic outcomes in OS 79. Given the pervasive presence of metabolically active CAFs across solid tumors, BHB likely represents a broader mechanism of metabolite-mediated immune suppression. The comprehensive profiling of CAF-derived metabolites may reveal additional immunosuppressive factors, thus providing insights into intertumoral variability in immunotherapy response and guiding the development of combinatorial metabolic-immunotherapeutic strategies.

This study has a few limitations. First, our conclusions are primarily based on NK-92 cell-based *in vitro* experiments and murine models. Although NK-92 cells provide a stable and reproducible system for NK-cell cytotoxicity, co-culture, metabolic assays, and genetic manipulation, their metabolic state, receptor repertoire, and cytotoxic properties may not fully represent primary NK cells derived from healthy donors or patients with osteosarcoma. Therefore, future studies using PBMC-derived NK cells, patient-derived peripheral blood NK cells, and tumor-infiltrating NK cells are needed to validate the clinical applicability of the CAF-BHB-FXR1 axis. In addition, the use of immunodeficient nude mice, while valuable for xenograft establishment, cannot fully recapitulate the complex interplay of the adaptive immune system. Further validation in species-matched immunocompetent models is required. Second, the present study did not include a sufficiently characterized clinical cohort or multivariable analysis; therefore, future studies should incorporate standardized BHB and NK-cell measurements, complete clinicopathological and multivariable validation in independent cohorts. Third, while we identified the BHB-FXR1 axis and its critical residue K56, our current evidence primarily demonstrates the necessity of this modification. In addition, although our data support the involvement of P300 in FXR1 Kbhb modification, P300 is a broad transcriptional coactivator and acyltransferase with important roles in cellular metabolic homeostasis. Although P300 knockdown increased FAO activity, consistent with the proposed inhibitory role of P300 in this pathway, the current experiments do not fully distinguish whether this effect is specifically mediated through FXR1 Kbhb or through broader transcriptional and acyltransferase functions of P300. Future studies using catalytically inactive P300 mutants, P300 rescue, or direct assessment of FXR1 Kbhb after P300 depletion will be required to establish the specific enzymatic dependence of this pathway. Similarly, the current SIRT2 findings are based on expression, interaction, overexpression, and promoter H3K9bhb enrichment; loss-of-function studies are needed to clarify its contribution to FXR1 deacetylation and Kbhb modification. To further establish a direct causal link, future studies should employ gain-of-function approaches, such as generating point mutants (e.g., K56Q) that mimic a constitutively Kbhb-modified state, to determine whether this modification alone is sufficient to drive NK cell dysfunction independently of BHB. Finally, the safety and efficacy of targeting this axis in preclinical and clinical settings require further investigation.

Future studies should validate these findings in clinically annotated independent cohorts and evaluate therapeutic strategies targeting BHB production or downstream signaling.

In conclusion, our study establishes a link between CAF metabolic activity and NK-cell suppression through a metabolic-epigenetic pathway, revealing a previously unrecognized immune evasion mechanism in OS.

## Supplementary Material

Supplementary figures.

## Figures and Tables

**Figure 1:**
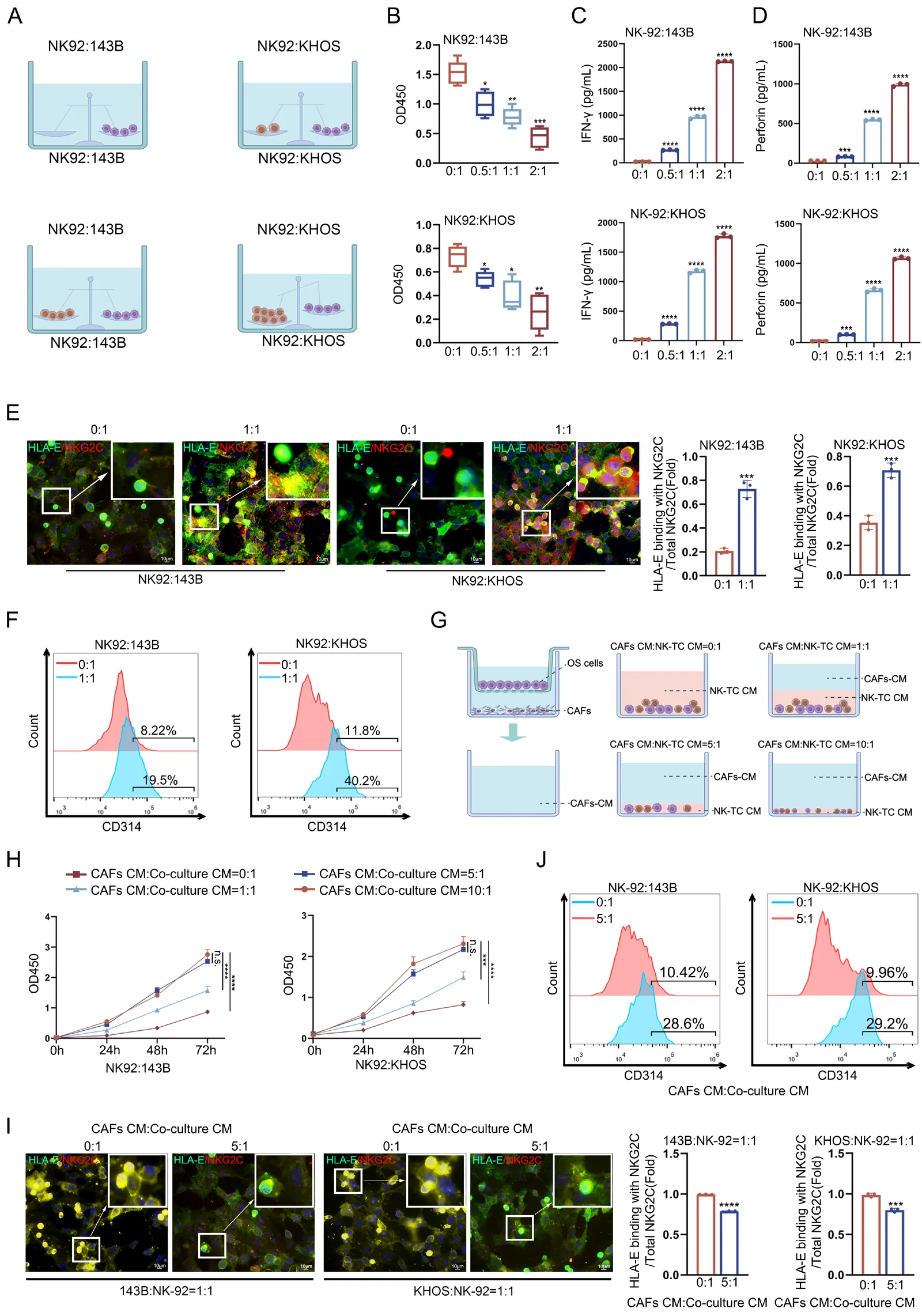
CAFs suppress NK cell-mediated cytotoxicity against osteosarcoma cells. (A) Schematic of the NK-osteosarcoma coculture model. (B) Proliferation of osteosarcoma cells assessed by CCK-8 assay. Data are presented as mean ± SD (n = 3 independent experiments). *p < 0.05, **p < 0.01, ***p < 0.001 by one-way ANOVA. (C) ELISA analysis comparing IFN-γ secretion levels in NK cells after treatment with NK-osteosarcoma supernatants. Data are presented as mean ± SD (n = 3 independent experiments). ****p < 0.0001 by one-way ANOVA. (D) ELISA analysis comparing perforin secretion levels in NK cells after treatment with NK-osteosarcoma supernatants. Data are presented as mean ± SD (n = 3 independent experiments). ***p < 0.001, ****p < 0.0001 by one-way ANOVA. (E) Immunofluorescence analysis of HLA-E expression in osteosarcoma cells and NKG2C expression in NK cells, showing colocalization at the tumor-NK interface. Data are presented as mean ± SD (n = 3 independent experiments). ***p < 0.001 by unpaired two-tailed Student's t-test. (F) Flow cytometric assessment of NK-cell activation. Data are presented as mean ± SD (n = 3 independent experiments). (G) Schematic of NK-tumor cocultures supplemented with supernatants from CAF-osteosarcoma cocultures. (H) Proliferation of osteosarcoma cells measured by CCK-8 assay. Data are presented as mean ± SD (n = 3 independent experiments). ***p < 0.001, ****p < 0.0001 by two-way ANOVA. (I) Immunofluorescence analysis of HLA-E/NKG2C colocalization after addition of CAF-osteosarcoma supernatants. Data are presented as mean ± SD (n = 3 independent experiments). ***p < 0.001, ****p < 0.0001 by unpaired two-tailed Student's t-test. (J) Flow cytometric analysis of NK-cell activation following CAF-osteosarcoma supernatant treatment. Data are presented as mean ± SD (n = 3 independent experiments).

**Figure 2:**
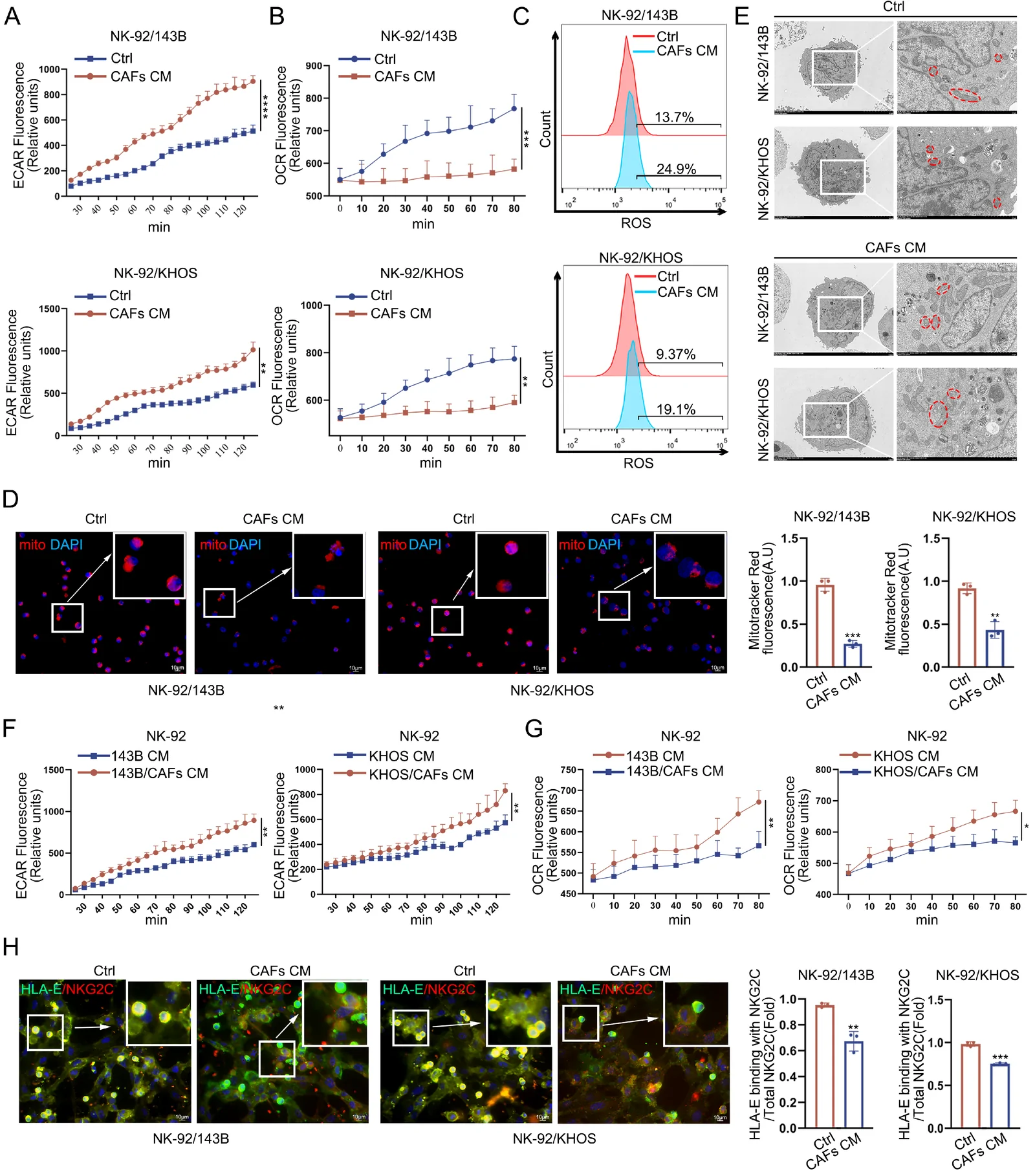
CAFs alter NK-cell metabolism and mitochondrial function. (A) Extracellular acidification rate (ECAR) of NK cells before and after treatment with CAF-conditioned medium (CM). Data are presented as mean ± SD (n = 3 independent experiments). **p < 0.01, ***p < 0.01 by two-way ANOVA. (B) Oxygen consumption rate (OCR) of NK cells following CAF-CM treatment. Data are presented as mean ± SD (n = 3 independent experiments). **p < 0.01 by two-way ANOVA. (C) Flow cytometric analysis of intracellular ROS activity in NK cells with or without CAF-CM treatment. Data are presented as mean ± SD (n = 3 independent experiments). (D) Immunofluorescence staining of NK cells using MitoTracker to assess mitochondrial integrity after CAF-CM exposure. Data are presented as mean ± SD (n = 3 independent experiments). **p < 0.01, ***p < 0.001 by unpaired two-tailed Student's t-test. (E) Transmission electron microscopy images showing mitochondrial ultrastructure in NK cells with or without CAF-CM treatment. (F) ECAR of NK cells treated with tumor cell-conditioned medium (tumor-CM) or conditioned medium from tumor cells co-cultured with CAFs (tumor/CAF-CM). Data are presented as mean ± SD (n = 3 independent experiments). **p < 0.01 by two-way ANOVA. (G) OCR of NK cells treated with tumor cell-conditioned medium (tumor-CM) or conditioned medium from tumor cells co-cultured with CAFs (tumor/CAF-CM). Data are presented as mean ± SD (n = 3 independent experiments). *p < 0.05, **p < 0.01 by two-way ANOVA. (H) Immunofluorescence analysis of HLA-E expression in osteosarcoma cells and NKG2C expression in NK cells, showing their colocalization after CAF-CM treatment. Data are presented as mean ± SD (n = 3 independent experiments). **p < 0.01, ***p < 0.001 by unpaired two-tailed Student's t-test.

**Figure 3:**
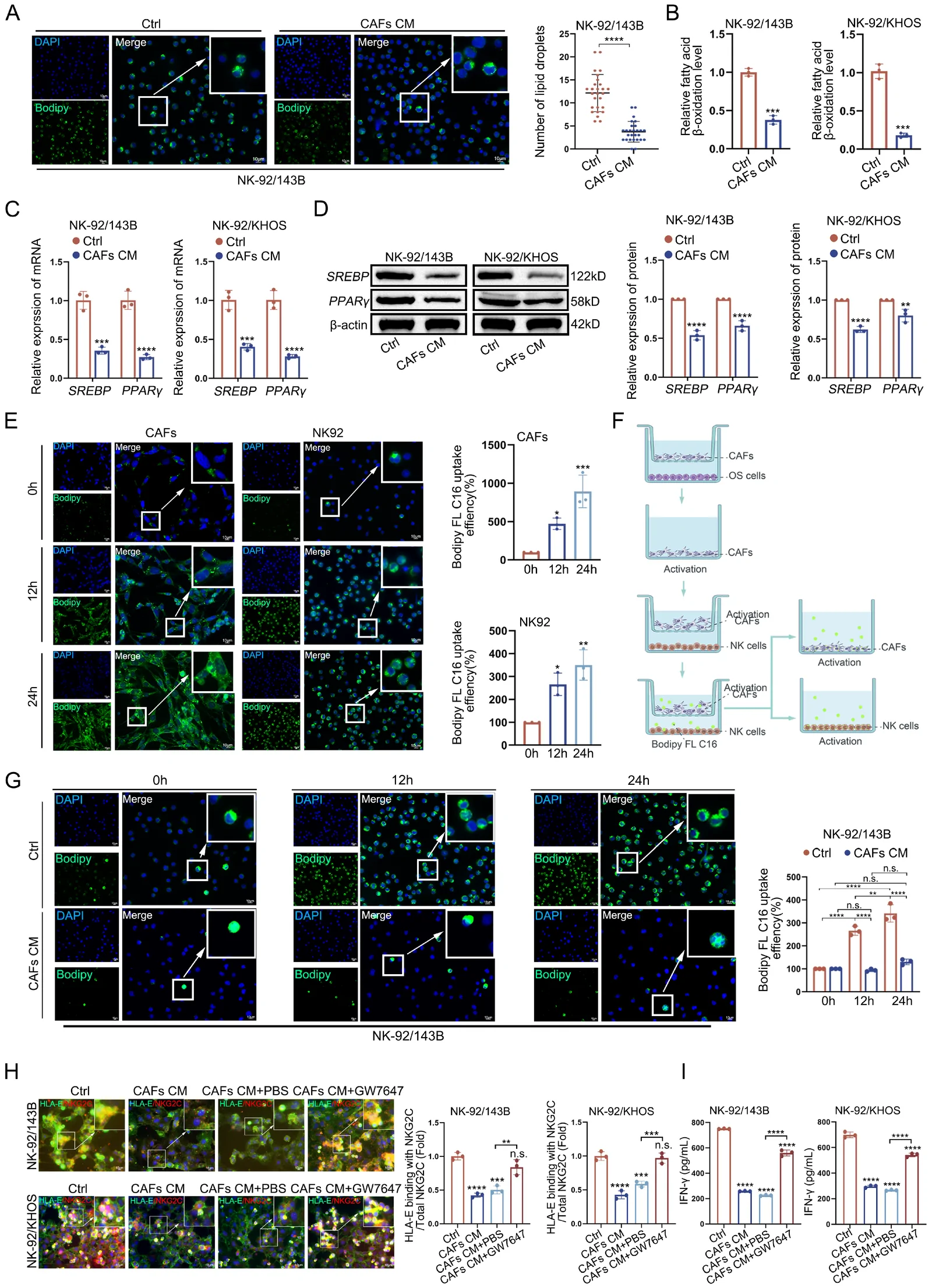
CAFs suppress NK-cell recognition of tumor cells by disrupting lipid metabolism. (A) BODIPY staining to assess intracellular lipid accumulation in NK cells following treatment with CAF-conditioned medium (CM). Data are presented as mean ± SD (n = 3 independent experiments). ****p < 0.0001 by unpaired two-tailed Student's t-test. (B) Fatty acid oxidation (FAO) activity of NK cells measured with a commercial FAO assay kit after CAF-CM treatment. Data are presented as mean ± SD (n = 3 independent experiments). ***p < 0.001 by unpaired two-tailed Student's t-test. (C) RT-qPCR analysis of FAO-related transcription factors *SREBP* and *PPARG* in NK cells with or without CAF-CM treatment. Data are presented as mean ± SD (n = 3 independent experiments). ***p < 0.001, ****p < 0.0001 by unpaired two-tailed Student's t-test. (D) Western blot analysis of *SREBP* and *PPARG* protein levels in NK cells after CAF-CM exposure. Data are presented as mean ± SD (n = 3 independent experiments). **p < 0.005, ****p < 0.0001 by unpaired two-tailed Student's t-test. (E) Quantification of lipid uptake in CAFs and NK cells cultured separately at 0, 12, and 24 h. Data are presented as mean ± SD (n = 3 independent experiments). *p < 0.05, **p < 0.01, ***p < 0.001 by one-way ANOVA. (F) Schematic of coculture experiments in which NK cells were incubated with CAFs in the presence of fluorescently labeled lipids. (G) Time-course analysis of lipid content in NK cells treated with CAF-CM at 0, 12, and 24 h. Data are presented as mean ± SD (n = 3 independent experiments). ^ns^p > 0.05, **p < 0.01, ****p < 0.0001 by two-way ANOVA. (H) Immunofluorescence analysis of HLA-E and NKG2C colocalization to assess NK cell recognition of osteosarcoma cells after treatment with CAF-CM in the presence or absence of the PPARα agonist GW7647. Data are presented as mean ± SD (n = 3 independent experiments). ^ns^p > 0.05, ***p < 0.01, ****p < 0.0001 by one-way ANOVA. (I) ELISA analysis of IFN-γ secretion levels in NK cells after treatment with CAF-CM in the presence or absence of GW7647. Data are presented as mean ± SD (n = 3 independent experiments). ****p < 0.0001 by one-way ANOVA.

**Figure 4:**
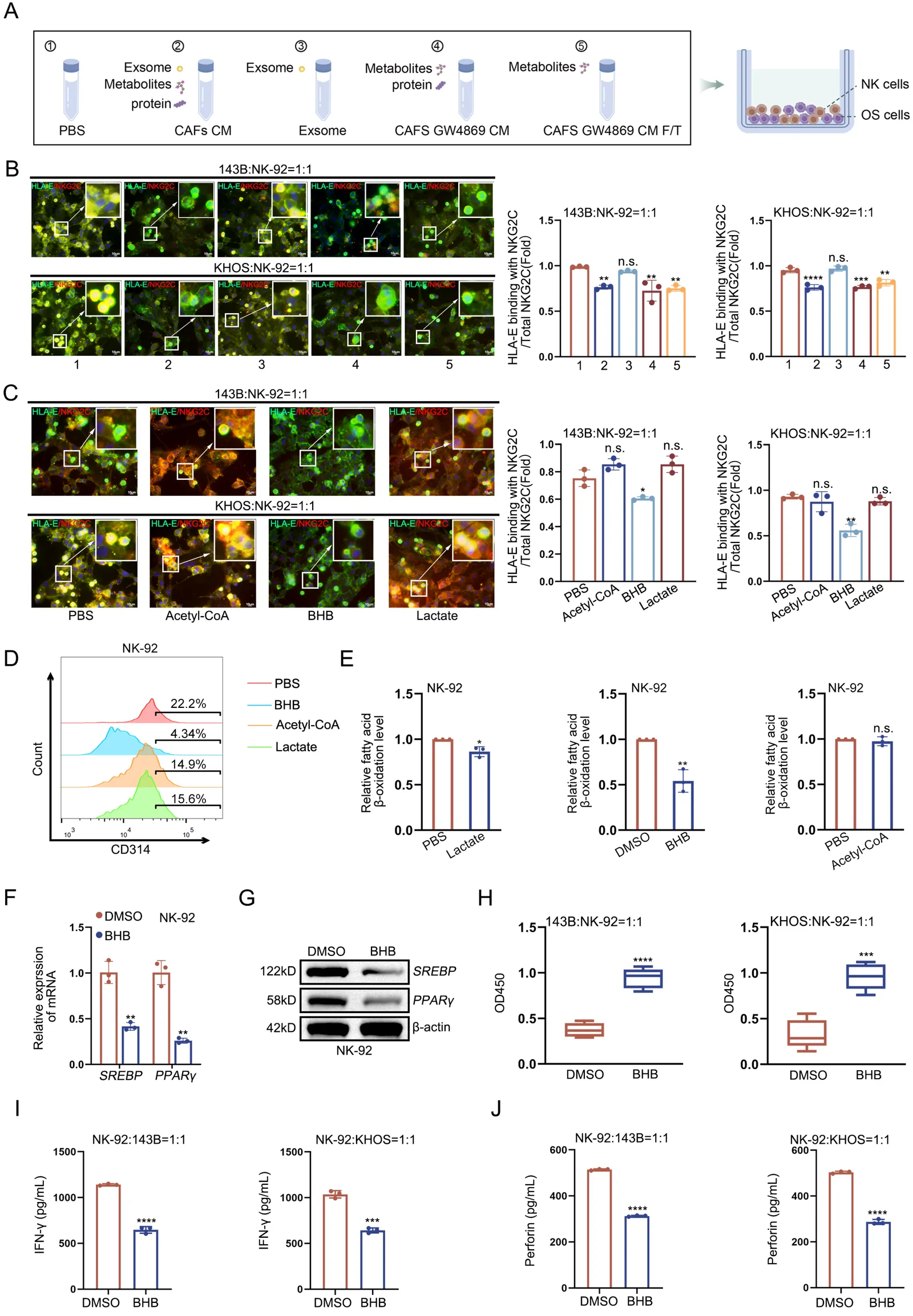
CAF-derived metabolite BHB impairs NK-cell lipid metabolism and suppresses tumor cell recognition. (A) Schematic of coculture experiments in which osteosarcoma cells and NK cells were treated with CAF-conditioned medium (CM), CAF-derived exosomes, CAF-CM with the exosome inhibitor GW4869, GW4869-treated triple freeze-thaw CAF-CM, or PBS control. (B) Immunofluorescence analysis of HLA-E expression on tumor cells and NKG2C expression on NK cells for evaluating receptor-ligand interactions. Data are presented as mean ± SD (n = 3 independent experiments). ^ns^p > 0.05, **p < 0.01, ***p < 0.001, ****p < 0.0001 by one-way ANOVA. (C) Immunofluorescence analysis of NK-cell activity following exposure to lipid metabolic intermediates including acetyl-CoA (100 μM), BHB (5 mM), and lactate (10 mM). Data are presented as mean ± SD (n = 3 independent experiments). ^ns^p > 0.05, *p < 0.05, **p < 0.01 by one-way ANOVA. (D) Flow cytometric analysis of NK-cell activation in response to individual lipid metabolites. (E) Assessment of FAO levels in NK cells following BHB treatment. Data are presented as mean ± SD (n = 3 independent experiments). ^ns^p > 0.05, *p < 0.05, **p < 0.01 by unpaired two-tailed Student's t-test. (F) RT-qPCR analysis of FAO-related genes *SREBP* and *PPARG* in BHB-treated NK cells. Data are presented as mean ± SD (n = 3 independent experiments). **p < 0.01 by one-way ANOVA. (G) Western blot validation of *SREBP* and *PPARG* expression in response to BHB. (H) CCK-8 assay assessing the killing capacity of NK cells against osteosarcoma cells after BHB treatment. Data are presented as mean ± SD (n = 3 independent experiments). ***p < 0.001, ****p < 0.0001 by unpaired two-tailed Student' s t-test. (I) ELISA analysis comparing IFN-γ expression levels in NK cells following BHB treatment. Data are presented as mean ± SD (n = 3 independent experiments). ***p < 0.001, ****p < 0.0001 by unpaired two-tailed Student' s t-test. (J) ELISA analysis comparing perforin expression levels in NK cells following BHB treatment. Data are presented as mean ± SD (n = 3 independent experiments). ****p < 0.0001 by unpaired two-tailed Student' s t-test.

**Figure 5:**
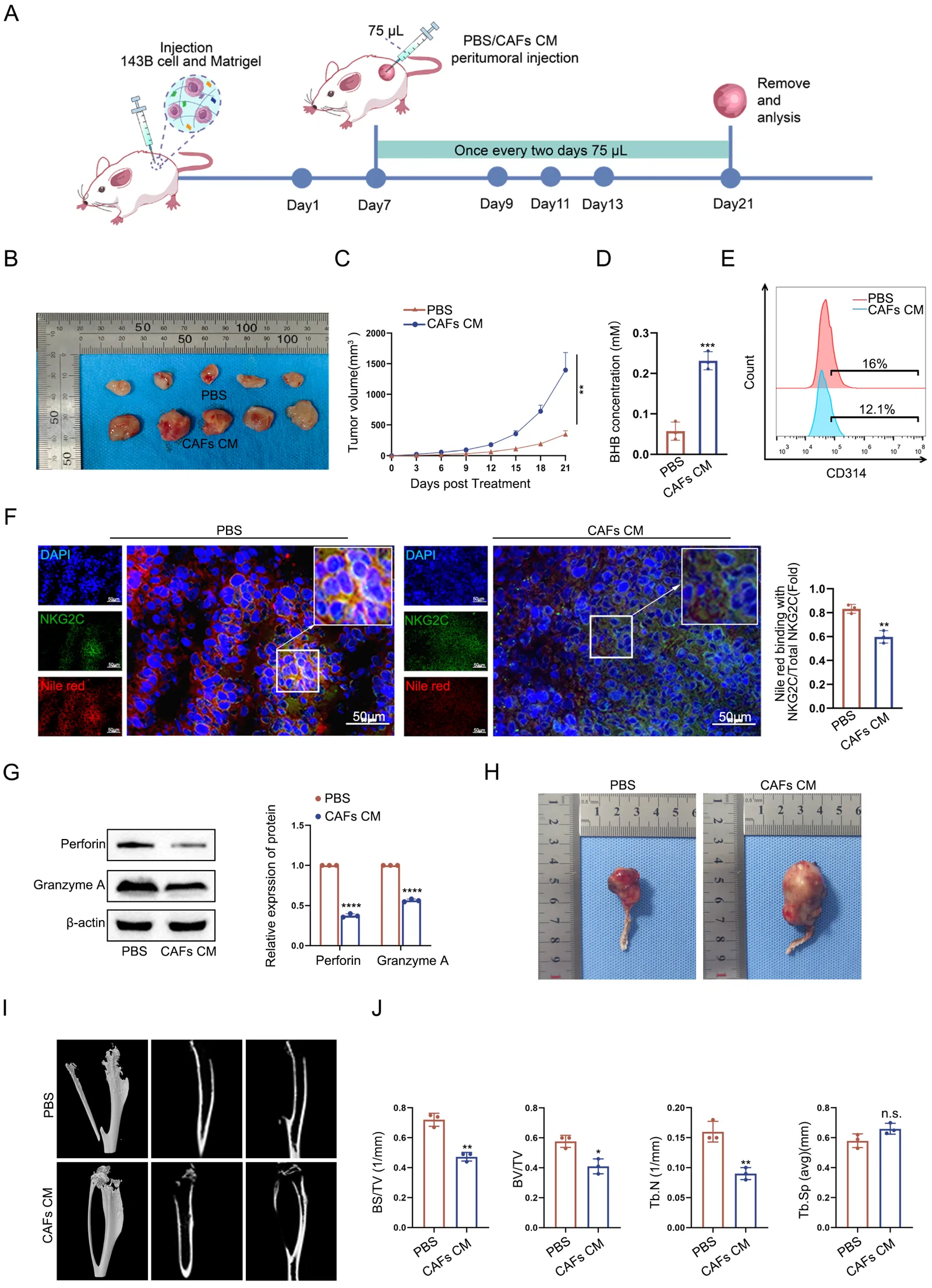
CAF-CM treatment is associated with tumor growth, circulating BHB, and NK-cell-related changes in nude mice. (A) Schematic illustration of a subcutaneous osteosarcoma xenograft model established in nude mice. (B) Representative macroscopic images of harvested subcutaneous tumors. n = 5 mice per group. (C) Measurement of tumor length and width for growth curve analysis. Data are presented as mean ± SD (n = 5 mice per group). **p < 0.01 by two-way ANOVA. (D) Quantification of circulating BHB levels in tumor-bearing mice. Data are presented as mean ± SD (n = 5 mice per group). ***p < 0.001 by unpaired two-tailed Student's t-test. (E) Flow cytometric analysis of CD314⁺ NK-cell activation in subcutaneous tumor tissues. (F) Nile red staining of lipid accumulation in intratumoral NK cells. Data are presented as mean ± SD (n = 5 mice per group). **p < 0.01 by unpaired two-tailed Student's t-test. (G) Western blot analysis of Granzyme A and perforin protein expression levels. Data are presented as mean ± SD (n = 3 independent experiments). ****p < 0.0001 by unpaired two-tailed Student's t-test. (H) Representative images of tumors. n = 5 mice per group. (I) Representative micro-CT images of proximal tibial cancellous bone. (J) Quantitative analysis of bone volume fraction (BV/TV), trabecular thickness (Tb.Th), trabecular number (Tb.N), and trabecular separation (Tb.Sp). Data are presented as mean ± SD (n = 3 independent experiments). ^ns^p > 0.05, *p < 0.05, **p < 0.01 by unpaired two-tailed Student's t-test.

**Figure 6:**
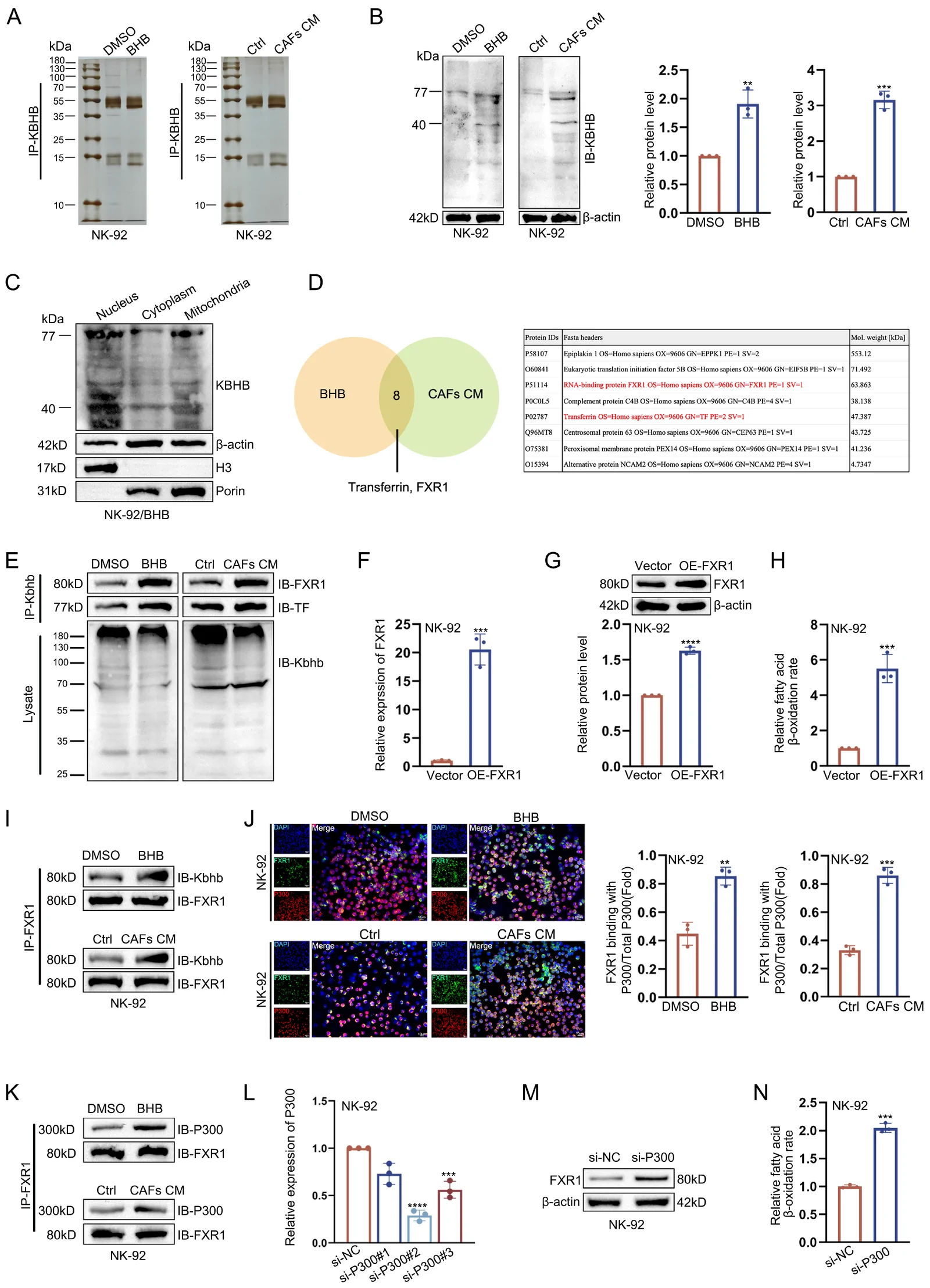
CAF-derived BHB promotes P300-associated Kbhb of FXR1 and suppresses fatty acid oxidation in NK cells. (A) Silver staining showing global Kbhb modifications in NK cells treated with CAF-CM or BHB, with enrichment of β-hydroxybutyrylated proteins by Kbhb-specific immunoprecipitation. (B) Western blot analysis of total Kbhb modification levels in NK cells following CAF-CM or BHB exposure. Data are presented as mean ± SD (n = 3 independent experiments). **p < 0.01, ***p < 0.001 by unpaired two-tailed Student's t-test. (C) Subcellular fractionation and western blot detection of Kbhb levels in cytoplasmic, nuclear, and mitochondrial proteins. (D) Proteomic profiling of Kbhb-enriched proteins in NK cells after CAF-CM or BHB treatment, with intersection analysis of identified candidates. (E) Western blot validation of transferrin and FXR1 among Kbhb-modified protein candidates. (F-G) RT-qPCR and western blot analysis to confirm FXR1 overexpression in NK cells. Data are presented as mean ± SD (n = 3 independent experiments). ***p < 0.001, ****p < 0.0001 by unpaired two-tailed Student's t-test. (H) Assessment of FAO levels in NK cells overexpressing FXR1. Data are presented as mean ± SD (n = 3 independent experiments). ***p < 0.001 by unpaired two-tailed Student's t-test. (I) Co-immunoprecipitation analysis of FXR1 Kbhb modification levels after treatment with CAF-CM or BHB. (J) Immunofluorescence colocalization of FXR1 with the Kbhb-modifying enzyme P300 after CAF-CM or BHB exposure. Data are presented as mean ± SD (n = 3 independent experiments). **p < 0.01, ***p < 0.001 by unpaired two-tailed Student's t-test. (K) Western blot analysis validating the interaction between FXR1 and P300 under CAF-CM or BHB treatment. (L-M) RT-qPCR and western blot confirmation of efficient P300 knockdown and its effect on FXR1 expression. Data are presented as mean ± SD (n = 3 independent experiments). ***p < 0.001, ****p < 0.0001 by one-way ANOVA. (N) Measurement of FAO activity in NK cells following P300 knockdown. P300 silencing significantly increased FAO activity compared with the si-NC group. Data are presented as mean ± SD (n = 3 independent experiments). ***p < 0.001 by unpaired two-tailed Student's t-test.

**Figure 7:**
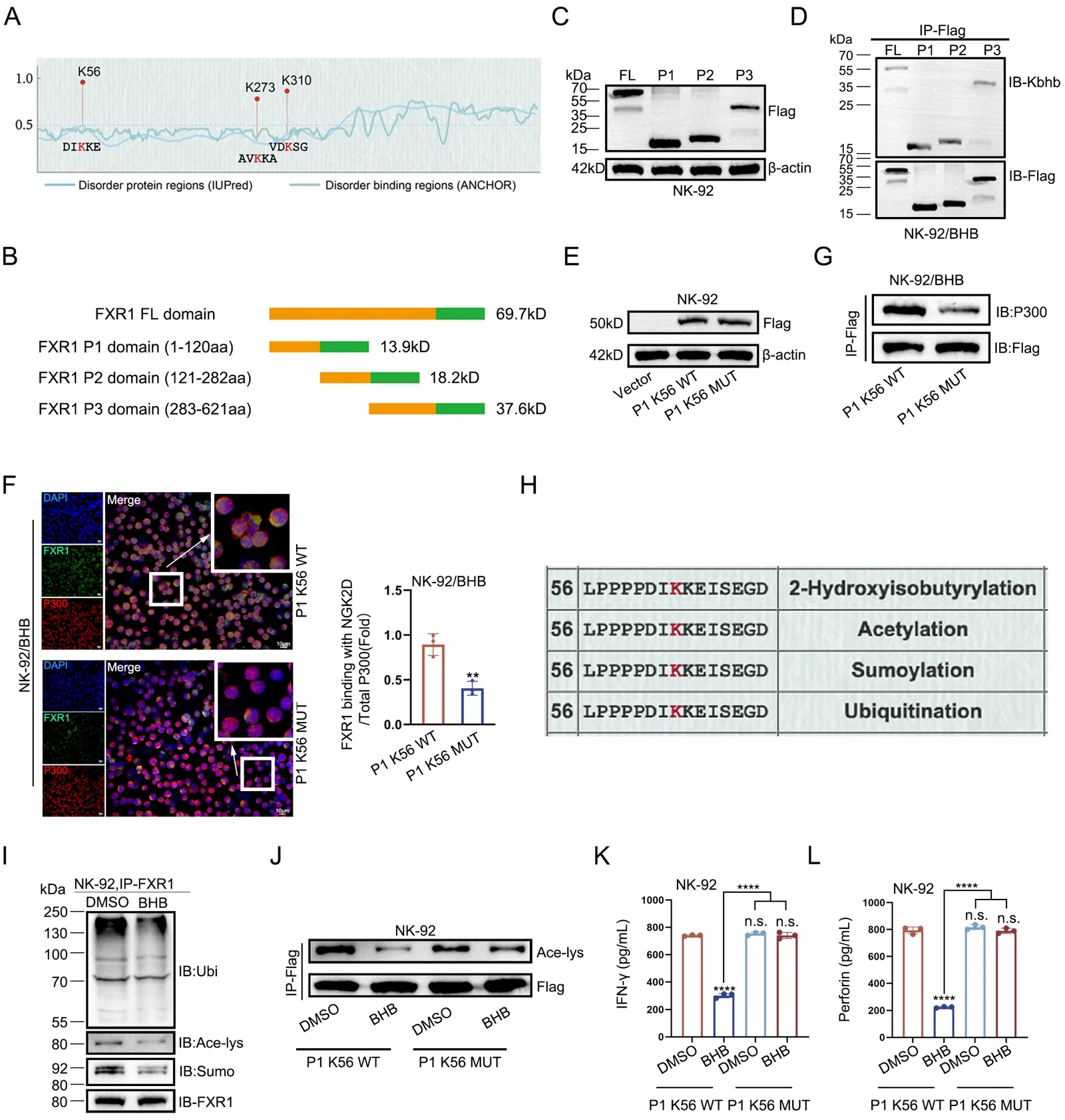
P300 mediates the shift of FXR1 modification at lysine 56 from acetylation to Kbhb. (A) Prediction of potential Kbhb modification sites on FXR1 using the pFunK database. (B) Schematic representation of FXR1 domain organization, including full length and residues 1-120, 121-282, and 283-621. (C) Western blot validation of FLAG-tagged FXR1 fusion protein expression in NK cells. (D) IP-FLAG analysis of BHB-treated NK cells showing domain-specific Kbhb modification and associated interacting proteins. (E) Western blot analysis of FXR1 K56 mutant fusion protein expression. (F) IF analysis of FXR1-P300 colocalization after K56 mutation. Data are presented as mean ± SD (n = 3 independent experiments). **p < 0.01 by unpaired two-tailed Student's t-test. (G) IP-western blot validation of the binding interaction between FXR1 and P300 following K56 mutation. (H) Posttranslational modification mapping at the FXR1 K56 site, indicating 2-Hydroxyisobutyrylation, acetylation, SUMOylation, and ubiquitination. (I) Western blot analysis of FXR1 modification dynamics following BHB treatment. (J) Western blot analysis comparing acetylation levels of wild-type and K56 mutant FXR1 after BHB treatment. (K) ELISA analysis comparing IFN-γ expression levels in wild-type and K56R mutant FXR1 after BHB treatment. Data are presented as mean ± SD (n = 3 independent experiments). ns, p < 0.001, ****p < 0.0001 by one-way ANOVA. (L) ELISA analysis comparing perforin expression levels in wild-type and K56R mutant FXR1 after BHB treatment. Data are presented as mean ± SD (n = 3 independent experiments). ns, p < 0.001, ****p < 0.0001 by one-way ANOVA.

**Figure 8:**
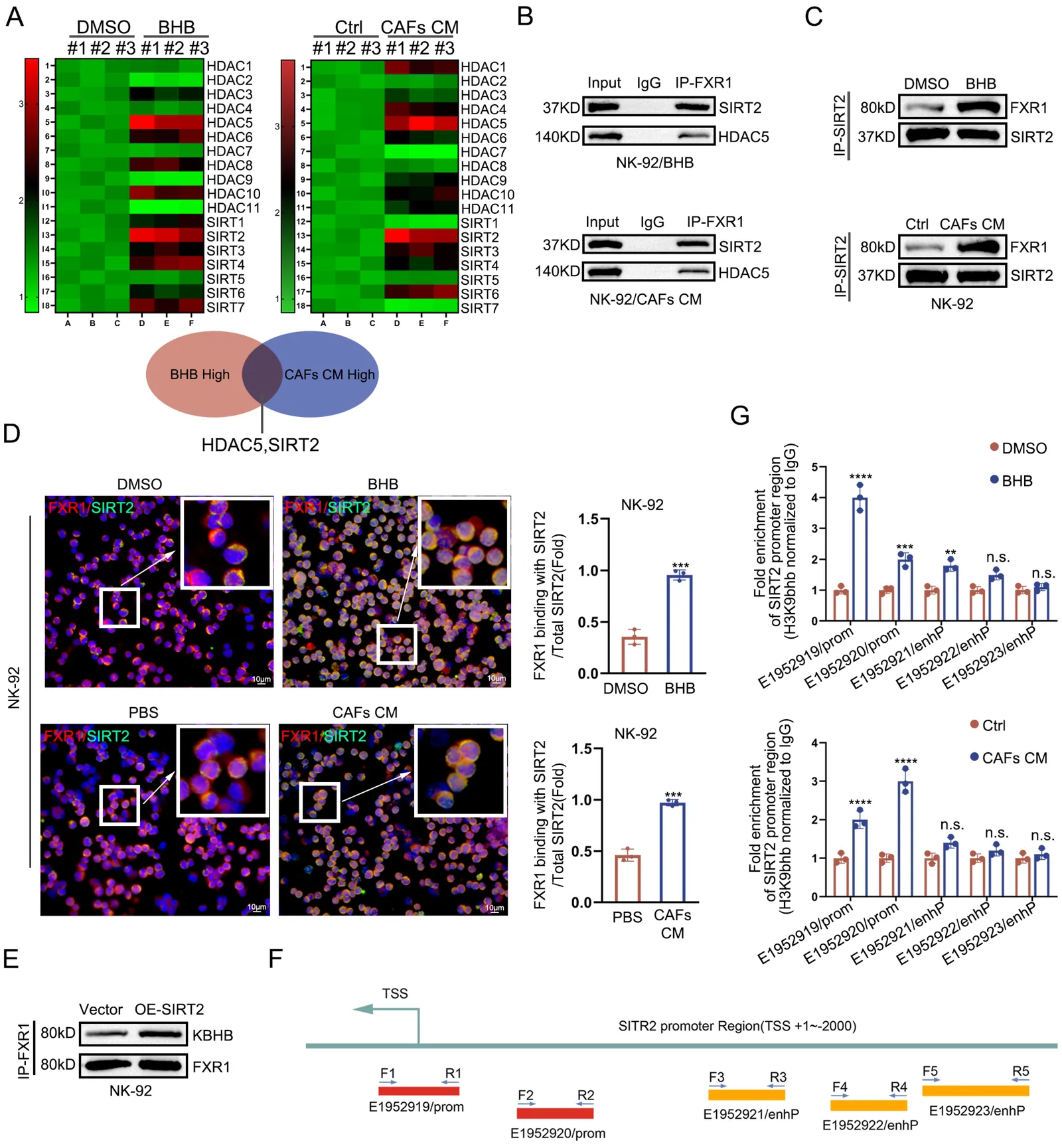
BHB is associated with H3K9bhb enrichment at the SIRT2 promoter and enhanced SIRT2-FXR1 interaction. (A) Heatmap of RT-qPCR analysis showing the effects of BHB on deacetylase expression, with HDAC5 and SIRT2 identified as upregulated targets. (B) Western blot analysis of FXR1 binding to HDAC5 and SIRT2 following treatment with CAF-conditioned medium or BHB. (C) Western blot detection of FXR1 enrichment in IP-SIRT2 protein complexes. (D) IF analysis of SIRT2-FXR1 colocalization after treatment with CAF-conditioned medium or BHB. Data are presented as mean ± SD (n = 3 independent experiments). ***p < 0.001 by unpaired two-tailed Student's t-test. (E) IP-western blot analysis of the effect of SIRT2 overexpression on FXR1 Kbhb modification in NK cells. (F) Primer design targeting the promoter activation region of the SIRT2 gene. (G) ChIP analysis of H3K9bhb modification levels in the SIRT2 promoter region after treatment with CAF-CM or BHB. Data are presented as mean ± SD (n = 3 independent experiments). ns, p < 0.001, **p < 0.01, ***p < 0.001, ****p < 0.0001 by one-way ANOVA.

**Table 1:** Plasmid sequences

Gene	Forward (5'-3')	Reverse (5'-3')
Flag-FXR1	AAGCTTATGGCGGAGCTGACGGTG	GGTACCTCAATCACATCTTTTGCCTAGCC
Flag-FXR1-P1	AAGCTTATGGCGGAGCTGACGGTG	GGTACCAGTTTTATTTTGATTGACAGGCCG
Flag-FXR1-P2	AAGCTTGTCAAAAAAAATACCTTCTTTAAATGCA	GGTACCATTTGTACCATAACCGGAGGTGTAA
Flag-FXR1-P3	AAGCTTGAGGATTTTATTCAGGTTCCTAGGAA	GGTACCTCAATCACATCTTTTGCCTAGCC
Flag-FXR1-P1 K56 MUT	CTGATATAAGAAAAGAAATTAGTGAAGGAGA	ATTTCTTTTCTTATATCAGGTGGTGGTGGTA
HA-SIRT2	GCTAGCATGGACTTCCTGCGGAACTTATT	AAGCTTTCACCCAGCCAGGCCACG

**Table 2:** siRNA sequences

Gene	Sense (5'-3')	Antisense (5'-3')
si-NC	UUCUCCGAACGUGUCACGUUU	ACGUGACACGUUCGGAGAAUU
si-FXR1#1	AGGCAAGGGAAGAGGCUAAUU	UUAGCCUCUUCCCUUGCCUUU
si-FXR1#2	AACUAAAGGUGGAGAAAGAUU	UCUUUCUCCACCUUUAGUUUU
si-FXR1#3	GCUGUAAGGUGUUAGCAAAUU	UUUGCUAACACCUUACAGCUU
si-P300#1	GGGAGUAAAUGGAGGUGUAUU	UACACCUCCAUUUACUCCCUU
si-P300#2	AGGAGGAAGAAGAGAGAAAUU	UUUCUCUCUUCUUCCUCCUUU
si-P300#3	CCAGAAAGAACUAGAAGAAUU	UUCUUCUAGUUCUUUCUGGUU
si-HMGCS2#1	CAAAUUUGGCCUCGGAGUAUU	UACUCCGAGGCCAAAUUUGUU
si-HMGCS2#2	UGGAGAAUGUGUAUGACUUUU	AAGUCAUACACAUUCUCCAUU
si-HMGCS2#3	GAACCCAUAUGGAGAAUGUUU	ACAUUCUCCAUAUGGGUUCUU

**Table 3:** RT-qPCR primer sequences

Gene	Forward (5'-3')	Reverse (5'-3')
*SREBP*	ACAGCCATGAAGACAGACGG	ATAGGCAGCTTCTCCGCATC
*PPARγ*	ACTTTGGGATCAGCTCCGTG	GCAGGCTCCACTTTGATTGC
FXR1	GATGATCGAGACAGCCGACA	TGTGATGAGATTCGCTGGCA
P300	CAGTCTGCCCCCAACCTAAG	GTTCTTTGCTTGCACCTGGG
HMGCS2	CTCCCTCTTCAATGCTGCCA	TAACATCGATCCAAGGCCCG
BDH1	GGCTTCCTTGTGTTTGCTGG	CAGACATTGAGCTGGACGGT
