# Supplementary material for: CAF-derived BHB modulates FXR1-Kbhb and NK-cell lipid metabolism in osteosarcoma
Source: Int J Biol Sci. 2026 Sep 2;22(14):7714–36. doi: 10.7150/ijbs.128764 (PMC13618007; doi:10.7150/ijbs.128764)
Supplement: Supplementary file 1 [file ijbsv22p7714s1.pdf]

Supplementary Figures

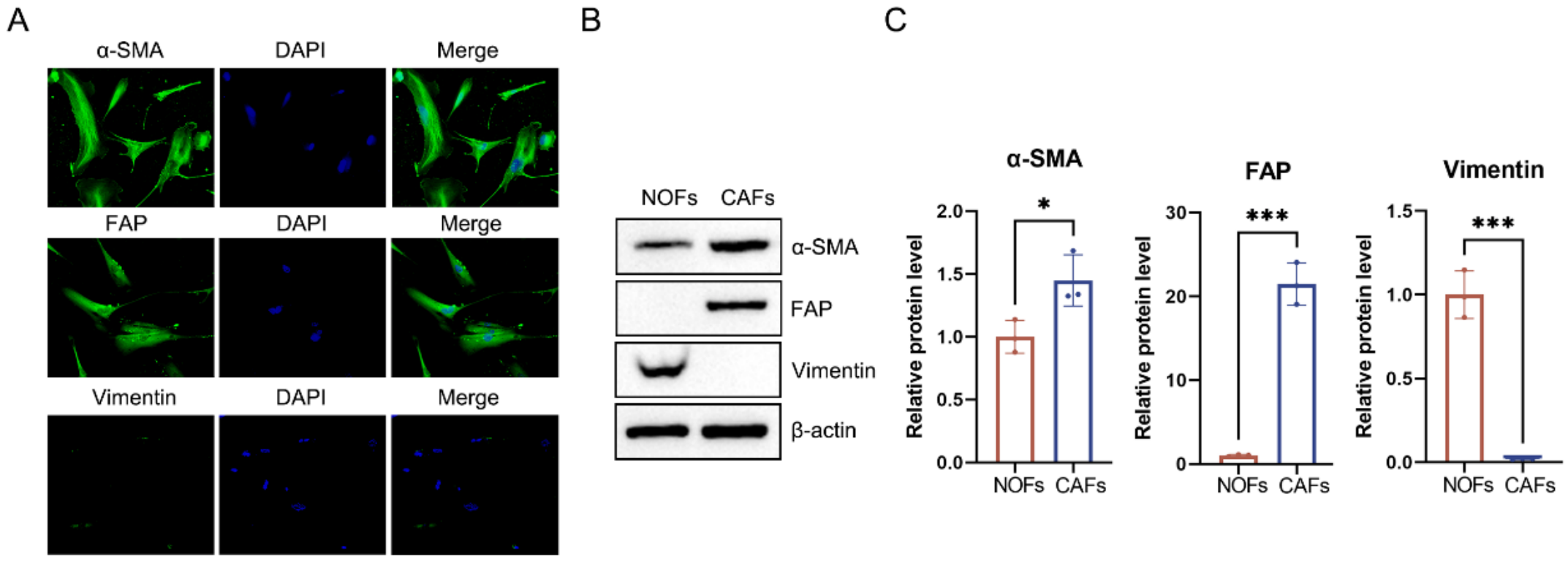

Figure S1. Characterization of isolated CAFs from osteosarcoma tissues. (A) Representative immunofluorescence images showing the expression of CAF-related markers α-SMA, FAP, and Vimentin in isolated CAFs. Nuclei were counterstained with DAPI. (B) Representative western blot images showing the protein expression of α-SMA, FAP, and Vimentin in NOFs and CAFs. β-actin was used as the loading control. (C) Quantitative analysis of α-SMA, FAP, and Vimentin protein levels in NOFs and CAFs. Data are presented as mean ± SD from three independent experiments. Statistical significance was determined using grouped *t*-test. *$P < 0.05$, ***$P < 0.001$.

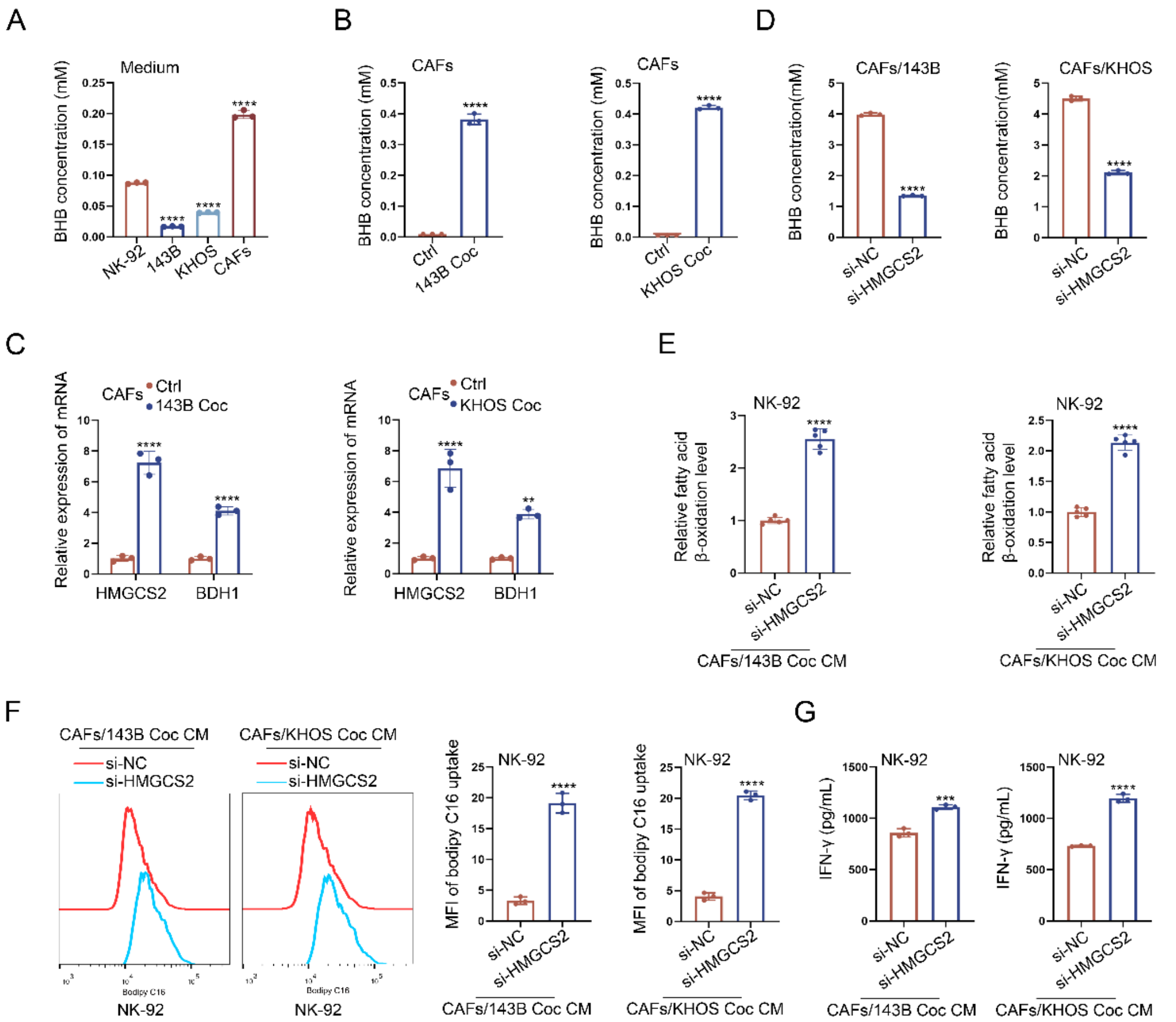

Figure S2. (A) Basal BHB levels in the supernatants of NK cells, osteosarcoma cells, and CAFs were measured by ELISA. Data are presented as mean ± SD (n = 3 independent experiments). ****p < 0.0001 by unpaired two-tailed Student's t-test. (B) BHB levels in the supernatants of CAFs after co-culture with osteosarcoma cells were measured by ELISA. Data are presented as mean ± SD (n = 3 independent experiments). ****p < 0.0001 by unpaired one-way ANOVA. (C) Expression levels of HMGCS2 and BDH1 in CAFs after co-culture with osteosarcoma cells were detected by RT-qPCR. Data are presented as mean ± SD (n = 3 independent experiments). **p < 0.01, ****p < 0.0001 by unpaired one-way ANOVA. (D) After co-culture with osteosarcoma cells, CAFs were transfected with si-HMGCS2 or si-NC, and BHB levels in CAF supernatants were measured by ELISA. Data are presented as mean ± SD (n = 3 independent experiments). ****p < 0.0001 by

unpaired two-tailed Student's t-test. (E) NK cells were treated with supernatants from CAFs transfected with si-NC or si-HMGCS2, and fatty acid oxidation levels in NK cells were measured. Data are presented as mean ± SD (n = 3 independent experiments). ****$p < 0.0001$ by unpaired two-tailed Student's t-test. (F) NK cells were treated with supernatants from CAFs transfected with si-NC or si-HMGCS2, and lipid uptake capacity in NK cells was detected by flow cytometry. Data are presented as mean ± SD (n = 3 independent experiments). ****$p < 0.0001$ by unpaired two-tailed Student's t-test. (G) NK cells were treated with supernatants from CAFs transfected with si-NC or si-HMGCS2, and IFN-γ secretion levels in NK cells were measured by ELISA. Data are presented as mean ± SD (n = 3 independent experiments). ***$p < 0.001$, ****$p < 0.0001$ by unpaired two-tailed Student's t-test.
